# Supplementary material for: The human hippocampus supports auditory coding and multisensory integration
Source: PLoS Biol. 2026 Nov 3;24(11):e3004012. doi: 10.1371/journal.pbio.3004012 (PMC13730386; doi:10.1371/journal.pbio.3004012)
Supplement: Supplementary file 1 [file pbio.3004012.s001.pdf]

Region	Visual		Auditory		Congruent AV		Incongruent AV	
	M (SEM)	P	M (SEM)	P	M (SEM)	P	M (SEM)	P
				Hippocampus				
**HPC**	0.088 (0.019)	<**0.001**	0.011 (0.019)	0.55	0.086 (0.018)	<**0.001**	0.071 (0.016)	<**0.001**
**aHPC**	0.088 (0.021)	<**0.001**	–0.003 (0.021)	0.89	0.092 (0.020)	<**0.001**	0.070 (0.019)	**0.001**
**pHPC**	0.086 (0.018)	<**0.001**	0.024 (0.019)	0.42	0.078 (0.018)	<**0.001**	0.073 (0.016)	<**0.001**
				Hippocampus by hemisphere				
**L. HPC**	0.085 (0.019)	<**0.001**	0.006 (0.019)	0.75	0.080 (0.018)	<**0.001**	0.066 (0.017)	<**0.001**
**R. HPC**	0.090 (0.019)	<**0.001**	0.016 (0.019)	0.75	0.091 (0.019)	<**0.001**	0.076 (0.017)	<**0.001**
**L. aHPC**	0.088 (0.022)	<**0.001**	–0.008 (0.021)	0.98	0.086 (0.019)	<**0.001**	0.065 (0.019)	**0.002**
**R. aHPC**	0.088 (0.023)	**0.001**	0.001 (0.021)	0.98	0.098 (0.022)	<**0.001**	0.073 (0.021)	**0.002**
**L. pHPC**	0.079 (0.018)	<**0.001**	0.018 (0.020)	0.38	0.074 (0.019)	<**0.001**	0.066 (0.016)	<**0.001**
**R. pHPC**	0.093 (0.019)	<**0.001**	0.031 (0.019)	0.22	0.082 (0.019)	<**0.001**	0.079 (0.016)	<**0.001**
				Hippocampal subfields				
**CA1**	0.052 (0.016)	**0.002**	0.006 (0.016)	0.71	0.046 (0.015)	**0.002**	0.038 (0.014)	**0.008**
**CA2/3**	0.090 (0.018)	<**0.001**	–0.009 (0.017)	0.71	0.076 (0.019)	<**0.001**	0.064 (0.019)	**0.003**
**DG**	0.109 (0.025)	<**0.001**	0.010 (0.024)	0.71	0.108 (0.025)	<**0.001**	0.086 (0.022)	**0.002**
**Subiculum**	0.105 (0.022)	<**0.001**	0.034 (0.023)	0.62	0.112 (0.022)	<**0.001**	0.100 (0.021)	<**0.001**
				MTL cortex				
**PHC**	0.299 (0.026)	<**0.001**	–0.061 (0.021)	**0.022**	0.320 (0.027)	<**0.001**	0.305 (0.022)	<**0.001**
**PRC**	0.064 (0.025)	**0.026**	–0.035 (0.024)	0.22	0.077 (0.024)	**0.005**	0.048 (0.025)	0.10
**EC**	0.030 (0.033)	0.37	–0.032 (0.034)	0.35	0.055 (0.036)	0.14	0.022 (0.035)	0.52
				Sensory cortex				
**OP**	0.996 (0.041)	<**0.001**	–0.190 (0.031)	<**0.001**	0.982 (0.041)	<**0.001**	0.966 (0.047)	<**0.001**
**LOC**	0.901 (0.032)	<**0.001**	–0.098 (0.021)	<**0.001**	0.892 (0.034)	<**0.001**	0.855 (0.034)	<**0.001**
**HG**	–0.332 (0.029)	<**0.001**	0.763 (0.038)	<**0.001**	0.708 (0.032)	<**0.001**	0.694 (0.033)	<**0.001**
**pSTG**	–0.087 (0.030)	**0.008**	0.282 (0.042)	<**0.001**	0.206 (0.039)	<**0.001**	0.211 (0.037)	<**0.001**

S1 Table. **Univariate activation relative to baseline.** M = mean β coefficient across participants. SEM = standard error of the mean. P = FDR-corrected statistical significance, bolded if $P < 0.05$.
