# Supplementary material for: The human hippocampus supports auditory coding and multisensory integration
Source: PLoS Biol. 2026 Nov 3;24(11):e3004012. doi: 10.1371/journal.pbio.3004012 (PMC13730386; doi:10.1371/journal.pbio.3004012)
Supplement: Supplementary file 2 [file pbio.3004012.s002.pdf]

Cluster	Region	Hem.	MNI (x, y, z)	Peak t	Size (mm^3)
			Auditory > visual		
1	Superior temporal gyrus (posterior)	R	$46, -19, -1$	20.52	317530
1a	Heschl's gyrus	L	$-49, -22, 9$	20.04	
1b	Heschl's gyrus	R	$47, -16, 3$	19.11	
1c	Planum temporale	L	$-51, -31, 7$	18.83	
2	Unlabeled	L	$-30, -95, -30$	5.61	3936
2a	Unlabeled	L	$-35, -88, -36$	5.53	
2b	Unlabeled	L	$-51, -77, -36$	5.30	
2c	Unlabeled	L	$-41, -85, -31$	5.08	
3	Postcentral gyrus	L	$-38, -24, 40$	5.60	44
4	Precentral gyrus	R	$58, -3, 47$	5.02	460
			Visual > auditory		
1	Thalamus	L	$-20, -31, 0$	25.34	877945
1a	Occipital fusiform gyrus	L	$-25, -84, -11$	23.37	
1b	Lingual gyrus	L	$-7, -90, -8$	22.07	
1c	Thalamus	R	$22, -31, 0$	22.01	
			Multisensory facilitation (congruent > auditory ∩ congruent > visual)		
1	Thalamus	R	$15, -26, -3$	10.68	5947
1a	Thalamus	L	$-18, -26, -5$	10.27	
1b	Thalamus	R	$14, -27, 4$	7.85	
1c	Brain-stem	L	$-6, -34, -6$	6.70	
2	Parietal operculum cortex	L	$-45, -31, 18$	6.62	5427
2a	Parietal operculum cortex	L	$-51, -37, 28$	6.44	
2b	Parietal operculum cortex	L	$-45, -34, 23$	6.27	
2c	Parietal operculum cortex	L	$-49, -28, 18$	5.98	
3	Parietal operculum cortex	R	$39, -27, 20$	6.27	5706
3a	Parietal operculum cortex	R	$49, -28, 21$	5.44	
3b	Parietal operculum cortex	R	$61, -32, 22$	4.87	
3c	Planum temporale	R	$45, -38, 16$	4.17	
4	Insular cortex	R	$37, -9, -5$	5.32	922
4a	Insular cortex	R	$37, 2, -19$	4.06	
4b	Insular cortex	R	$38, 1, -10$	3.60	
4c	Temporal pole	R	$27, 4, -23$	3.51	
5	Insular cortex	L	$-35, -8, 13$	4.85	1434
5a	Insular cortex	L	$-36, -17, -7$	4.55	
5b	Insular cortex	L	$-38, -8, -12$	4.55	
5c	Insular cortex	L	$-37, -15, -7$	4.30	
6	Supramarginal gyrus (posterior)	L	$-53, -43, 13$	3.63	376
6a	Planum temporale	L	$-48, -46, 19$	2.47	
7	Brain-stem	L	$-11, -31, -24$	3.59	54
8	Temporal pole	L	$-34, 2, -17$	3.55	159
8a	Temporal pole	L	$-32, 6, -28$	2.91	
9	Frontal orbital cortex	R	$36, 31, -25$	3.44	285
10	Insular cortex	R	$30, -29, 8$	3.37	70
11	Frontal orbital cortex	R	$52, 29, -20$	3.32	77
12	Precentral gyrus	R	$61, -5, 24$	3.06	306
12a	Postcentral gyrus	R	$62, -5, 15$	2.39	
13	Angular gyrus	R	$61, -48, 14$	2.55	182
			Congruent > incongruent		
1	Paracingulate gyrus	L	$-6, 50, -2$	7.01	3382
1a	Paracingulate gyrus	L	$-7, 42, -10$	5.90	
1b	Frontal medial cortex	L	$-9, 51, -6$	5.84	
1c	Frontal medial cortex	R	$9, 40, -15$	5.41	

Continued on next page

S2 Table (continued)

Cluster	Region	Hem.	MNI (x, y, z)	Peak t	Size (mm^3)
2	Lateral occipital cortex (inferior)	R	$57, -68, -6$	4.54	38
			Incongruent > congruent		
1	Superior parietal lobule	L	$-38, -57, 45$	6.28	1381
2	Inferior frontal gyrus (pars opercularis)	L	$-41, 15, 24$	6.07	4278
2a	Middle frontal gyrus	L	$-53, 18, 29$	5.60	
2b	Inferior frontal gyrus (pars triangularis)	L	$-54, 24, 23$	5.37	
2c	Middle frontal gyrus	L	$-36, 5, 33$	5.19	
3	Middle frontal gyrus	R	$52, 22, 29$	5.78	512
3a	Middle frontal gyrus	R	$42, 23, 22$	5.60	

S2 Table. **List of peak activations in the whole-brain GLM analysis.** Significant clusters were identified using threshold-free cluster enhancement (TFCE) at $P < 0.05$. Bidirectional contrasts are reported in both directions (auditory vs. visual; congruent vs. incongruent). Anatomical labels were assigned to each peak using the Harvard-Oxford atlas (Desikan et al., 2006). For each cluster, the peak and any local maxima at least 8 mm apart (labeled a, b, c) are shown. Clusters smaller than 20 voxels are omitted.
