# Supplementary material for: The human hippocampus supports auditory coding and multisensory integration
Source: PLoS Biol. 2026 Nov 3;24(11):e3004012. doi: 10.1371/journal.pbio.3004012 (PMC13730386; doi:10.1371/journal.pbio.3004012)
Supplement: Supplementary file 3 [file pbio.3004012.s004.pdf]

Region	Visual		Auditory		Congruent AV	
	M (SEM)	P	M (SEM)	P	M (SEM)	P
			Hippocampus			
**HPC**	0.002 (0.001)	0.11	0.002 (0.001)	**0.017**	0.004 (0.001)	**0.001**
**aHPC**	–0.000 (0.002)	0.51	0.004 (0.001)	**0.007**	0.003 (0.001)	**0.012**
**pHPC**	0.003 (0.001)	**0.031**	0.000 (0.001)	0.46	0.005 (0.002)	**0.003**
			Hippocampus by hemisphere			
**L. HPC**	0.001 (0.002)	0.36	0.002 (0.001)	0.072	0.004 (0.002)	**0.026**
**R. HPC**	0.002 (0.002)	0.24	0.003 (0.001)	0.058	0.005 (0.001)	**0.001**
**L. aHPC**	–0.000 (0.002)	0.54	0.003 (0.002)	0.066	0.002 (0.002)	0.20
**R. aHPC**	–0.000 (0.002)	0.54	0.004 (0.002)	**0.013**	0.005 (0.002)	**0.010**
**L. pHPC**	0.001 (0.002)	0.33	0.000 (0.002)	0.55	0.006 (0.002)	**0.011**
**R. pHPC**	0.004 (0.002)	**0.039**	–0.000 (0.002)	0.55	0.004 (0.002)	**0.036**
			Hippocampal subfields			
**CA1**	0.002 (0.002)	0.19	0.002 (0.002)	0.24	0.002 (0.002)	0.19
**CA2/3**	0.000 (0.003)	0.43	0.002 (0.003)	0.36	0.001 (0.002)	0.24
**DG**	0.000 (0.002)	0.43	0.000 (0.002)	0.40	0.004 (0.002)	**0.031**
**Subiculum**	0.002 (0.002)	0.19	0.004 (0.002)	0.088	0.006 (0.003)	**0.031**
			MTL cortex			
**PHC**	0.045 (0.003)	$<$**0.001**	0.006 (0.002)	**0.002**	0.045 (0.003)	$<$**0.001**
**PRC**	0.001 (0.002)	0.33	0.001 (0.002)	0.54	0.003 (0.002)	**0.027**
**EC**	–0.000 (0.003)	0.55	–0.002 (0.003)	0.69	0.002 (0.003)	0.32
			Sensory cortex			
**OP**	0.061 (0.008)	$<$**0.001**	0.006 (0.004)	0.084	0.071 (0.006)	$<$**0.001**
**LOC**	0.090 (0.020)	$<$**0.001**	0.021 (0.008)	**0.004**	0.085 (0.009)	$<$**0.001**
**HG**	0.009 (0.002)	$<$**0.001**	0.083 (0.005)	$<$**0.001**	0.088 (0.005)	$<$**0.001**
**pSTG**	0.025 (0.005)	$<$**0.001**	0.053 (0.004)	$<$**0.001**	0.067 (0.006)	$<$**0.001**

S3 Table. **Multivariate pattern similarity relative to baseline.** M = mean movie-specific pattern similarity across participants. SEM = standard error of the mean. P = FDR-corrected statistical significance, bolded if $P < 0.05$.
