# Supplementary material for: The human hippocampus supports auditory coding and multisensory integration
Source: PLoS Biol. 2026 Nov 3;24(11):e3004012. doi: 10.1371/journal.pbio.3004012 (PMC13730386; doi:10.1371/journal.pbio.3004012)
Supplement: Supplementary file 4 [file pbio.3004012.s005.pdf]

Region	**Congruent > Visual**		**Congruent > Auditory**		**Crossmodal Transfer**	
	ΔM (SEM)	P	ΔM (SEM)	P	M (SEM)	P
			Hippocampus			
**HPC**	0.003 (0.001)	**0.029**	0.002 (0.002)	0.15	0.002 (0.001)	**0.003**
**aHPC**	—	—	—	—	0.003 (0.001)	**0.003**
**pHPC**	0.002 (0.001)	0.084	0.005 (0.002)	**0.013**	0.001 (0.001)	0.22
			Hippocampus by hemisphere			
**L. HPC**	0.003 (0.002)	0.099	0.002 (0.003)	0.23	0.001 (0.001)	0.26
**R. HPC**	0.003 (0.001)	0.074	0.002 (0.002)	0.23	0.003 (0.001)	**0.006**
**L. aHPC**	—	—	—	—	0.001 (0.001)	0.20
**R. aHPC**	—	—	—	—	0.004 (0.002)	**0.016**
**L. pHPC**	0.005 (0.002)	**0.009**	0.005 (0.003)	**0.040**	0.000 (0.001)	0.49
**R. pHPC**	—	—	—	—	0.002 (0.001)	0.16
			Hippocampal subfields			
**CA1**	—	—	—	—	0.000 (0.001)	0.42
**CA2/3**	—	—	—	—	0.002 (0.001)	0.14
**DG**	0.004 (0.003)	0.065	0.004 (0.002)	0.074	0.002 (0.001)	0.091
**Subiculum**	0.004 (0.002)	0.065	0.002 (0.004)	0.34	0.002 (0.001)	0.091
			MTL cortex			
**PHC**	—	—	—	—	0.008 (0.001)	<**0.001**
**PRC**	0.002 (0.002)	0.28	0.003 (0.003)	0.25	0.002 (0.001)	0.14
**EC**	0.002 (0.005)	0.35	0.003 (0.004)	0.25	0.001 (0.002)	0.39
			Sensory cortex			
**OP**	0.010 (0.008)	0.13	0.065 (0.006)	<**0.001**	0.005 (0.002)	**0.020**
**LOC**	—	—	—	—	0.017 (0.013)	**0.047**
**HG**	0.079 (0.005)	<**0.001**	0.005 (0.002)	**0.025**	0.004 (0.001)	**0.004**
**pSTG**	0.042 (0.005)	<**0.001**	0.014 (0.005)	**0.006**	0.009 (0.003)	**0.004**

S4 Table. **Multisensory effects.** Multivariate evidence for multisensory facilitation (congruent > visual; congruent > auditory) and crossmodal transfer. $(\Delta)M$ = (difference in) mean movie-specific pattern similarity across participants. SEM = standard error of the mean. P = FDR-corrected statistical significance, bolded if < 0.05. Facilitation was tested only in regions where congruent numerically exceeded both unisensory conditions (— = not tested).
