# Supplementary material for: The human hippocampus supports auditory coding and multisensory integration
Source: PLoS Biol. 2026 Nov 3;24(11):e3004012. doi: 10.1371/journal.pbio.3004012 (PMC13730386; doi:10.1371/journal.pbio.3004012)
Supplement: Supplementary file 5 [file pbio.3004012.s006.pdf]

Region	β_A		β_V		R^2		Residual MSPS	
	M (SEM)	P	M (SEM)	P	M (SEM)	P	M (SEM)	P
Hippocampus								
**HPC**	–0.008 (0.013)	0.72	0.053 (0.021)	**0.003**	–0.005 (0.007)	0.68	0.004 (0.001)	**0.002**
**aHPC**	0.013 (0.014)	0.36	0.016 (0.011)	0.082	–0.008 (0.002)	1.00	0.003 (0.001)	**0.019**
**pHPC**	–0.019 (0.014)	0.90	0.051 (0.021)	**0.005**	–0.008 (0.007)	1.00	0.005 (0.002)	**0.005**
Sensory cortex								
**OP**	0.049 (0.019)	**0.009**	0.344 (0.032)	<**0.001**	0.108 (0.023)	<**0.001**	0.044 (0.005)	<**0.001**
**LOC**	0.039 (0.027)	0.079	0.316 (0.041)	<**0.001**	0.122 (0.028)	<**0.001**	0.050 (0.006)	<**0.001**
**HG**	0.565 (0.031)	<**0.001**	0.075 (0.026)	**0.003**	0.358 (0.040)	<**0.001**	0.037 (0.002)	<**0.001**
**pSTG**	0.283 (0.034)	<**0.001**	0.113 (0.034)	<**0.001**	0.108 (0.024)	<**0.001**	0.045 (0.005)	<**0.001**

S5 Table. **Unisensory contributions to congruent audiovisual similarity.** Cross-validated multiple regression modeling the congruent audiovisual similarity matrix as a function of the auditory-only and visual-only similarity matrices. β_A, β_V = auditory-only and visual-only regression coefficients. R^2 = proportion of variance in the congruent audiovisual matrix explained by the unisensory matrices on held-out folds. Residual MSPS = movie-specific pattern similarity in the congruent audiovisual condition remaining after regressing out the unisensory contributions. M = mean across participants. SEM = standard error of the mean. P = FDR-corrected statistical significance, bolded if < 0.05. R^2 was tested one-tailed against zero; P values approaching 1 therefore indicate cross-validated variance explained reliably below zero.
