# Supplementary material for: The human hippocampus supports auditory coding and multisensory integration
Source: PLoS Biol. 2026 Nov 3;24(11):e3004012. doi: 10.1371/journal.pbio.3004012 (PMC13730386; doi:10.1371/journal.pbio.3004012)
Supplement: Supplementary file 6 [file pbio.3004012.s017.pdf]

Cluster	Region	Hem.	MNI (x, y, z)	Peak t	Size (mm^3)
			Auditory > visual		
1	Planum temporale	R	59, −8, 2	13.22	30042
1a	Heschl's gyrus	R	45, −24, 9	12.36	
1b	Heschl's gyrus	R	53, −14, 6	11.09	
1c	Central opercular cortex	R	50, −19, 12	10.36	
2	Heschl's gyrus	L	−51, −14, 2	12.96	19472
2a	Heschl's gyrus	L	−53, −21, 7	11.67	
2b	Planum temporale	L	−58, −15, 4	10.69	
2c	Heschl's gyrus	L	−45, −24, 7	10.46	
3	Superior frontal gyrus	R	17, 23, 45	6.18	8041
3a	Superior frontal gyrus	L	−1, 37, 44	6.06	
3b	Superior frontal gyrus	R	9, 30, 44	5.69	
3c	Superior frontal gyrus	L	−16, 27, 39	5.60	
4	Inferior frontal gyrus (pars opercularis)	R	57, 18, 13	4.13	31
5	Inferior frontal gyrus (pars triangularis)	R	52, 25, 18	3.94	143
			Visual > auditory		
1	Lingual gyrus	R	26, −51, −8	19.21	530125
1a	Occipital fusiform gyrus	R	32, −73, −8	18.82	
1b	Occipital fusiform gyrus	R	38, −67, −10	18.18	
1c	Occipital fusiform gyrus	R	28, −73, −7	17.80	
2	Temporal fusiform cortex (posterior)	R	40, −10, −38	4.98	172
3	Brain-stem	R	16, −27, −33	4.15	180
4	Unlabeled	L	−7, −62, −38	4.09	387
4a	Unlabeled	L	−14, −66, −37	2.86	
4b	Unlabeled	L	−13, −58, −32	2.84	
4c	Unlabeled	L	−8, −58, −43	2.76	
5	Temporal fusiform cortex (posterior)	R	40, −9, −29	3.64	143
6	Temporal fusiform cortex (anterior)	R	34, −6, −36	3.40	60
7	Brain-stem	R	3, −16, −16	3.04	106
8	Putamen	L	−22, 0, 7	3.03	40
			Multisensory facilitation (congruent > auditory ∩ congruent > visual)		
1	Planum temporale	R	62, −30, 19	9.12	24725
1a	Planum temporale	R	51, −29, 14	8.13	
1b	Planum temporale	R	64, −21, 16	8.10	
1c	Superior temporal gyrus (posterior)	R	61, −31, 11	7.89	
2	Superior temporal gyrus (posterior)	L	−63, −36, 12	7.76	35829
2a	Parietal operculum cortex	L	−52, −33, 20	7.62	
2b	Planum temporale	L	−60, −35, 12	7.33	
2c	Parietal operculum cortex	L	−39, −35, 22	7.22	
3	Unlabeled	L	−33, −51, 9	3.67	255
4	Insular cortex	R	37, 14, −10	3.42	56
5	Inferior frontal gyrus (pars opercularis)	R	40, 16, 23	2.91	119
6	Amygdala	L	−28, −3, −14	2.75	35
7	Insular cortex	L	−39, 13, −9	2.68	32
8	Temporal pole	R	45, 12, −13	2.46	57
			Crossmodal transfer		
1	Planum temporale	R	59, −33, 14	9.92	237255
1a	Lateral occipital cortex (inferior)	R	49, −61, 14	9.62	
1b	Lateral occipital cortex (inferior)	R	52, −65, 7	9.30	
1c	Angular gyrus	R	51, −58, 15	9.17	
2	Precentral gyrus	R	48, 0, 47	6.56	324
3	Superior frontal gyrus	L	−8, 1, 67	6.05	25
4	Middle frontal gyrus	L	−32, 1, 40	3.66	629

Continued on next page

S6 Table (continued)

**Cluster**	**Region**	**Hem.**	MNI (x, y, z)	**Peak** t	**Size (mm^3)**
4a	Middle frontal gyrus	L	-34, 10, 36	3.49	
4b	Middle frontal gyrus	L	-31, 7, 28	3.04	
4c	Middle frontal gyrus	L	-34, 6, 39	2.95	
			Unisensory > incongruent		
1	Superior temporal gyrus (posterior)	R	65, -33, 14	10.54	330533
1a	Lateral occipital cortex (inferior)	R	53, -62, 8	9.15	
1b	Planum temporale	R	55, -36, 19	8.77	
1c	Angular gyrus	R	61, -49, 15	8.61	

S6 Table. **List of peak activations in the whole-brain RSA searchlight analysis.** Significant clusters were identified using threshold-free cluster enhancement (TFCE) at $P < 0.05$. Bidirectional contrasts are reported in both directions (auditory vs. visual). Anatomical labels were assigned to each peak using the Harvard-Oxford atlas (Desikan et al., 2006). For each cluster, the peak and any local maxima at least 8 mm apart (labeled a, b, c) are shown. Clusters smaller than 20 voxels are omitted.
