# Supplementary material for: The human hippocampus supports auditory coding and multisensory integration
Source: PLoS Biol. 2026 Nov 3;24(11):e3004012. doi: 10.1371/journal.pbio.3004012 (PMC13730386; doi:10.1371/journal.pbio.3004012)
Supplement: Supplementary file 7 [file pbio.3004012.s020.pdf]

Response to Reviewers

Editor's note

As you will see below, the reviewers are overall quite positive, but nevertheless raise a few concerns that require additional analyses and textual revisions to expand the discussion of quite a few points.

Thank you for the opportunity to revise our manuscript. We carefully addressed the Reviewers' comments through a series of new analyses, resulting in a new figure in the main text and 9 new figures and tables in the supplement. We report new evidence of hemispheric lateralization of multisensory representations and of conjunctive audiovisual coding in the hippocampus. We also expand our treatment of retrieval-based accounts of our findings and of hippocampal long-axis specialization. Finally, we refined our anterior/posterior hippocampal segmentations and re-ran all analyses to verify the robustness of our pipeline. The manuscript has been revised to reflect the results of all these new and updated analyses.

Reviewer #1

In this manuscript, Raccah and colleagues examine whether the human hippocampus represents sensory input beyond vision and supports cross-modal representations. After a pre-scanner study phase, a relatively large sample of participants (N=30) viewed short movie clips across four conditions: visual only, auditory only, congruent auditory and visual stimuli, and incongruent auditory and visual stimuli. The authors then analyzed BOLD activity within sub-regions of hippocampus (anterior vs posterior and subfields) using both univariate and multivariate pattern similarity approaches They test whether hippocampal ROIs: 1) represent auditory input, 2) are sensitive to multisensory facilitation, and 3) support cross-modal representations.

They find that univariate responses in the hippocampus are robust for visual stimuli but show no significant modulation for auditory or audio-visual conditions. In contrast, multivariate pattern analyses reveal both auditory and visual representations, with significant cross-modal pattern similarity. This cross-modal similarity is driven by the anterior hippocampus (i.e., not posterior), which shows evidence for crossmodal generalization between auditory and visual stimuli. Overall, this is a great manuscript: well written, technically strong, and focused on a question of broad relevance. The results are compelling, particularly the evidence for cross-modal pattern similarity in the anterior hippocampus. My main suggestions concern interpretation. In particular, the manuscript would benefit from a fuller treatment of retrieval-based alternative accounts

of the results, clearer explanation of the control analyses, and more discussion of whether the auditory-only and visual-only conditions may differ in cognitive processes (e.g., imagery, attention) in addition to the input modality itself. Specific comments follow.

We thank the Reviewer for their thoughtful feedback and supportive comments on our manuscript.

Major:

1. Apologies if I missed this, but what, if anything, was presented on the screen during the auditory-only condition?

Thank you for pointing out this omission. During auditory-only trials, participants fixated on a central cross that remained on the screen for the full 6-s trial and remained on the screen during the inter-trial interval. The fixation cross was also present during visual-only and audiovisual trials, superimposed over the videos. We have now clarified this in the revision:

Results (p. 2): “A central fixation cross remained on screen throughout each 6-s trial across all conditions, superimposed over videos in the visual-only and audiovisual conditions.”

Methods (p. 13): “A central fixation cross was presented for the full 6 s of every trial, including during auditory-only trials, and was superimposed over videos in the visual-only and audiovisual conditions.”

2. The authors run a control analysis on p5 to determine whether the multivariate results could in principle come from memory retrieval. They write: "The multivariate effects reported above could in principle benefit from memory retrieval, with repeated stimulus exposure allowing for reinstatement of movie-specific details or mental imagery of the absent modality. If so, we would expect that the magnitudes of these effects should increase as stimuli are repeated across cross-validation folds. However, neither multisensory facilitation in the posterior hippocampus (congruent audiovisual >visual MSPS; Spearman's $\rho = 0.048$, $P = 0.45$) nor crossmodal transfer in the anterior hippocampus ($\rho = 0.05$, $P = 0.41$) strengthened with more stimulus repetition across runs. This lack of correlation suggests that both phenomena were relatively stable and fails to support a retrieval-based account." This is an important issue to address, but the logic of the analysis is not yet clear to me. What exactly was done here? As written, it is difficult to understand how "stimulus repetition across cross-validation folds" was

quantified, and why this analysis provides a strong test of a retrieval-based account. A more straightforward approach would be to split the runs in half and ask whether the relevant multivariate effects are stronger in the second half of the experiment than in the first, when repeated exposure should have had more opportunity to strengthen retrieval or imagery-based contributions.

We apologize for the lack of clarity in our original description. Our main RSA uses leave-one-run-out cross-validation, in which each run is held out and correlated with the average of the remaining runs. This yields 9 estimates (one per fold), each corresponding to a run's position in the task. We reasoned that the contribution of retrieval or imagery should grow with repeated exposure and therefore produce larger effects in folds where later runs were held out. We therefore labeled the cross-validation folds by held-out run position (runs 1–9) and correlated run position with the magnitude of our effects of interest (multisensory facilitation and crossmodal transfer) in that run. If repeated exposure strengthened retrieval- or imagery-based contributions, we would expect a positive correlation between run position and effect magnitude. We have now clarified this logic in the revised text (p. 6):

"The multivariate effects reported above could in principle benefit from memory retrieval, with repeated stimulus exposure allowing for reinstatement of movie-specific details or mental imagery of the absent modality. If so, we would expect that the magnitude of these effects should increase across repeated stimulus presentations. We tested this possibility in two complementary analyses. First, we leveraged the leave-one-run-out cross-validation RSA procedure, in which each run is held out and correlated with the average of the remaining runs. This yields 9 estimates (one per fold), each corresponding to a run's position in the task. We therefore labeled the cross-validated folds by held-out run position (runs 1–9) and correlated run position with the magnitude of our effects of interest (multisensory facilitation and crossmodal transfer) in that run. If repeated exposure strengthened retrieval- or imagery-based contributions, we would expect a positive correlation between run position and effect magnitude."

We agree with the Reviewer that a split-half analysis is a straightforward and complementary approach for evaluating a retrieval-based account. Inspired by their comment, we ran this new analysis by computing cross-validated RSA separately within the first four runs and the last four runs (omitting the middle fifth run to balance the comparison). Within each half, we estimated multisensory facilitation in the posterior hippocampus (congruent audiovisual > visual-only MSPS) and crossmodal transfer in the anterior hippocampus. We observed a trend toward diminished multisensory facilitation in pHPC and enhanced crossmodal transfer in aHPC in the later runs (see

below). However, neither effect differed significantly between the two halves (paired randomization test, 10,000 iterations).

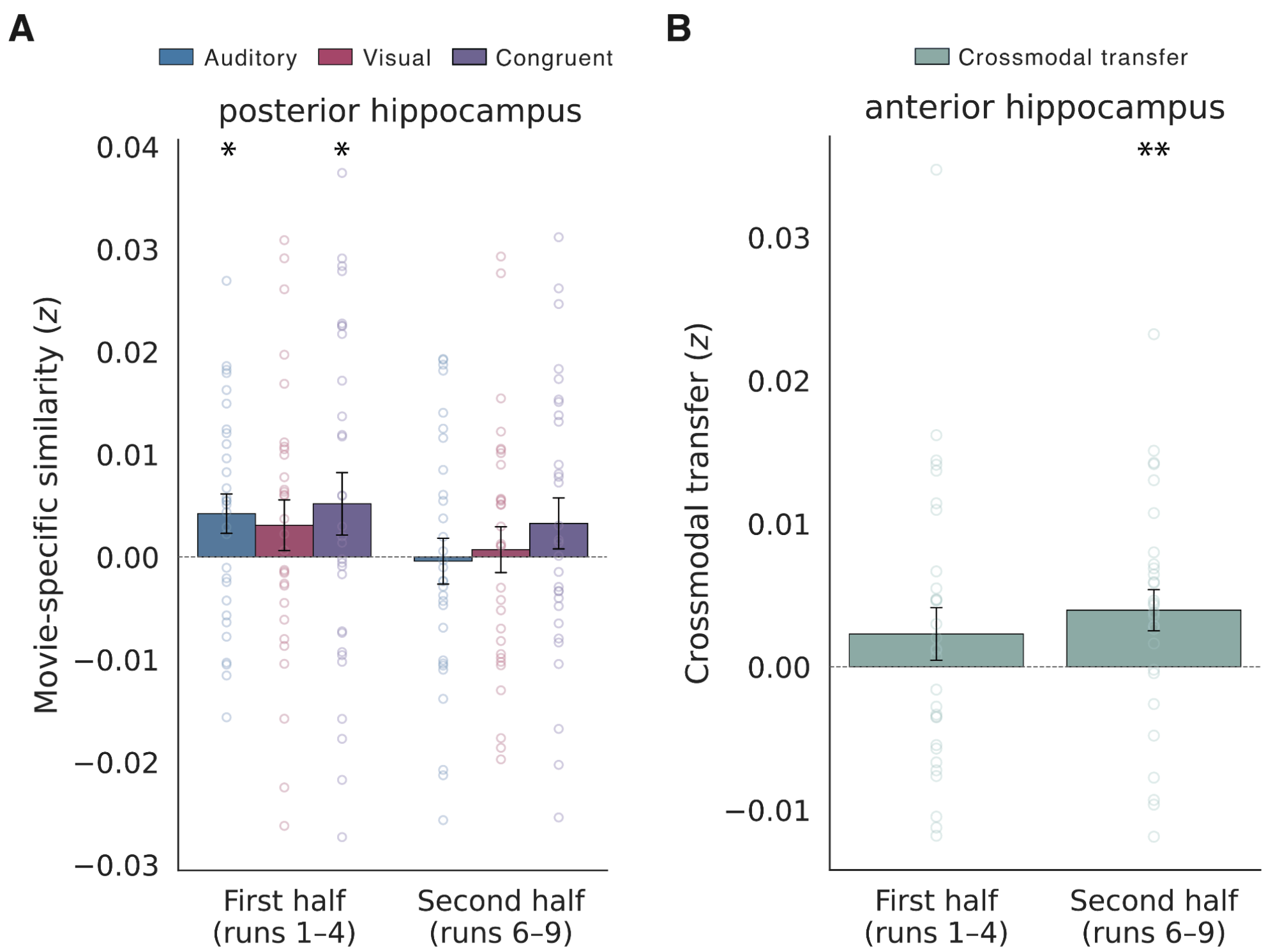

Figure S10: **Split-half analysis of multisensory facilitation and crossmodal transfer. (A)** MSPS for each condition in the posterior hippocampus, estimated separately within the first half (runs 1–4) and second half (runs 6–9) of the experiment. **(B)** Crossmodal transfer MSPS in the anterior hippocampus across the same two halves. Individual participant data shown as circles; error bars indicate SEM. * $P < 0.05$, ** $P < 0.01$.

We have added this figure to the revised supplement (Figure S10) and report this analysis in the revised manuscript (p. 6-7):

"Second, we ran a split-half analysis comparing effects estimated within the first four runs versus the last four runs (omitting the middle run for balance; Figure S10). In the first half, the posterior hippocampus showed significant MSPS for the auditory-only (P = 0.017) and congruent audiovisual (P = 0.050) conditions; in the second half, there were no significant effects. In contrast, crossmodal transfer in the anterior hippocampus was significant in the second half (P = 0.005) but not the first (P = 0.12). However, neither multisensory facilitation nor crossmodal transfer differed significantly between the two halves (both $P > 0.05$). These results raise the possibility that facilitation and transfer might be affected differently by stimulus repetitions, perhaps because crossmodal transfer involves learning amodal abstractions."

3. Of course, even if such an analysis shows no increase over runs, it would still be possible that retrieval processes were already at ceiling in run 1 because of the pre-scanner study phase. The authors should therefore more fully consider and discuss this alternative account of their findings.

We agree and have included a paragraph in the revised Discussion to emphasize this possibility and propose future work that could adjudicate between perceptual and retrieval-based accounts (p. 11):

“Although our analyses across repeated exposures provided limited support for a retrieval-based account of these effects, we observed a trend toward enhanced crossmodal transfer in later runs, which suggests that these effects may be driven in part by learned audiovisual associations (Figure S10). In fact, even without an increase across runs, it is possible that the pre-experimental exposure phase enabled retrieval processes even early in the experiment.”

We also connect this interpretation to our discussion of hippocampal long-axis specialization (p. 11-12):

“A more recent network-driven account instead links the posterior hippocampus to salience and target detection, and the anterior hippocampus to memory and scene construction (Angeli et al., 2025). This is broadly consistent with our analyses across repeated exposures, which showed a trend toward stronger crossmodal transfer in the anterior hippocampus (Figure S10)”

4. Univariate results: Given that a primary goal is to determine whether hippocampal voxels show significant univariate modulation to auditory-only versus video-only movie clips, it is important to clearly establish that any differences between these conditions are driven by input modality rather than by differences in the cognitive processes they engage. Two such processes come to mind. First, imagery or recall. Because the audiovisual clips were paired in memory through both pre-scanner practice and in-scanner presentation, hearing an audio clip alone - for example, the sound of someone cutting a cucumber - may automatically evoke visual imagery of the corresponding scene. The reverse may also occur, with visual input eliciting auditory imagery, but my intuition is that this process may be asymmetric, with auditory input more likely to trigger visual imagery than vice versa. If so, the two conditions may differ not only in sensory modality, but also in the extent to which they recruit internally generated imagery. One possible way to address this may be to ask whether a reactivation index (e.g., % activity during audio-only in V1 vs. visual-only in V1)

calculated for each condition is matched. At minimum, the authors should discuss a retrieval-based account of their findings.

Thank you for this comment. We agree it is important to establish the extent to which each modality elicits activity in sensory regions of the opposing modality. As suggested, we extracted univariate GLM estimates from four sensory ROIs (Harvard-Oxford atlas): lower- and higher-level visual regions (occipital pole [OP] and lateral occipital cortex [LOC], respectively) and two auditory regions (Heschl's gyrus [HG] and posterior superior temporal gyrus [pSTG]). If visual processing is engaged during auditory presentation, we expect above-baseline activity in the visual areas (OP and LOC) for the auditory-only condition; conversely, if auditory processing is engaged during visual presentation, we expect above-baseline activity in the auditory areas (HG and pSTG) for the visual-only condition. As shown below, we did not observe significant above-baseline signal for the opposing modality in these ROIs; if anything, they were suppressed below baseline:

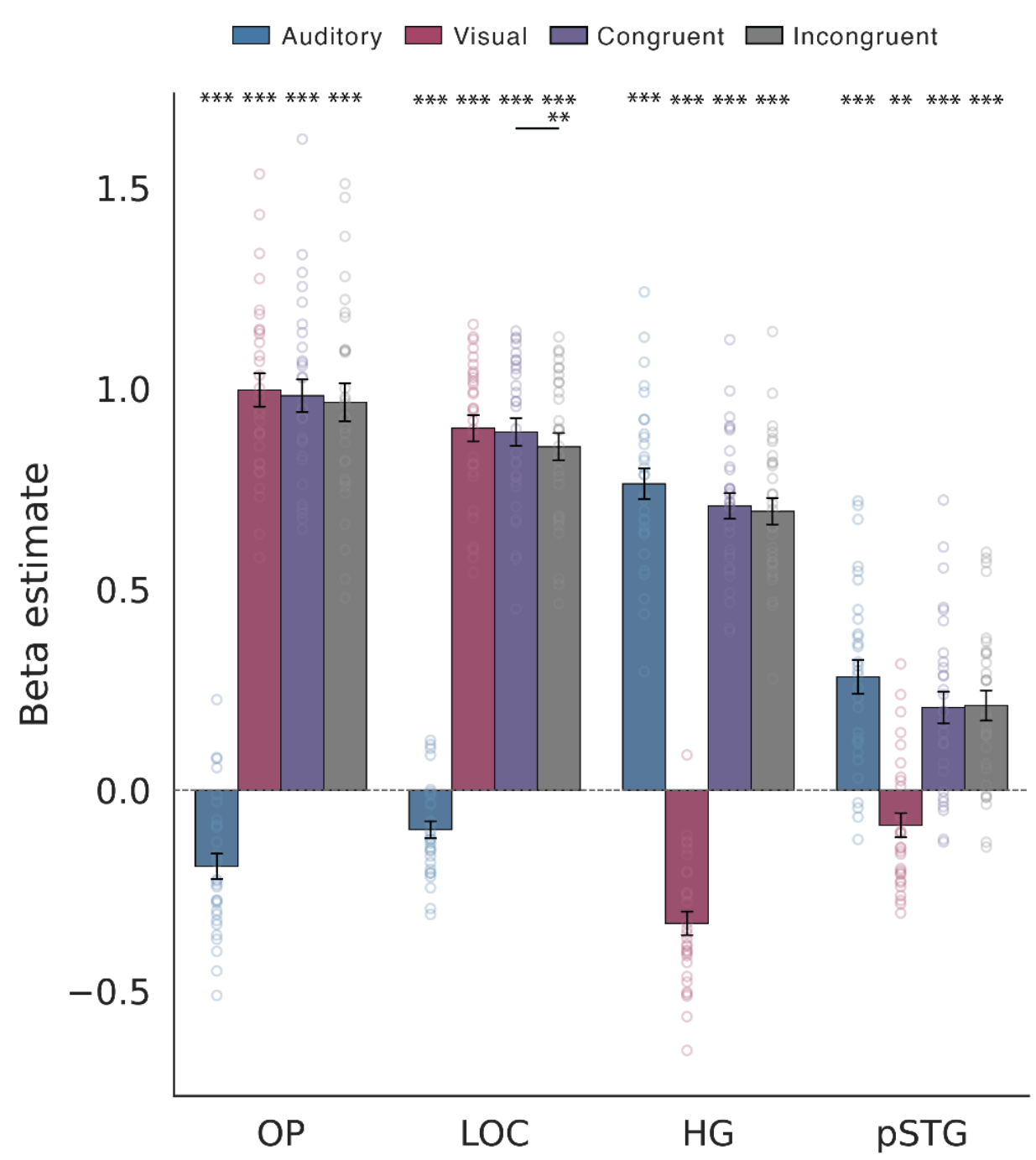

Univariate GLM results in sensory ROIs. Mean beta estimates for each condition in sensory cortical ROIs (OP, LOC, HG, pSTG). Individual participant data shown as circles; error bars indicate SEM. ** *P* < 0.01, *** *P* < 0.001.

Although the univariate analysis did not indicate reliable mean activation, reinstatement could be captured in multivariate patterns. We therefore computed MSPS in these

cortical ROIs. We found that LOC (but not OP) showed auditory-only MSPS, and that HG and pSTG showed significant visual-only MSPS:

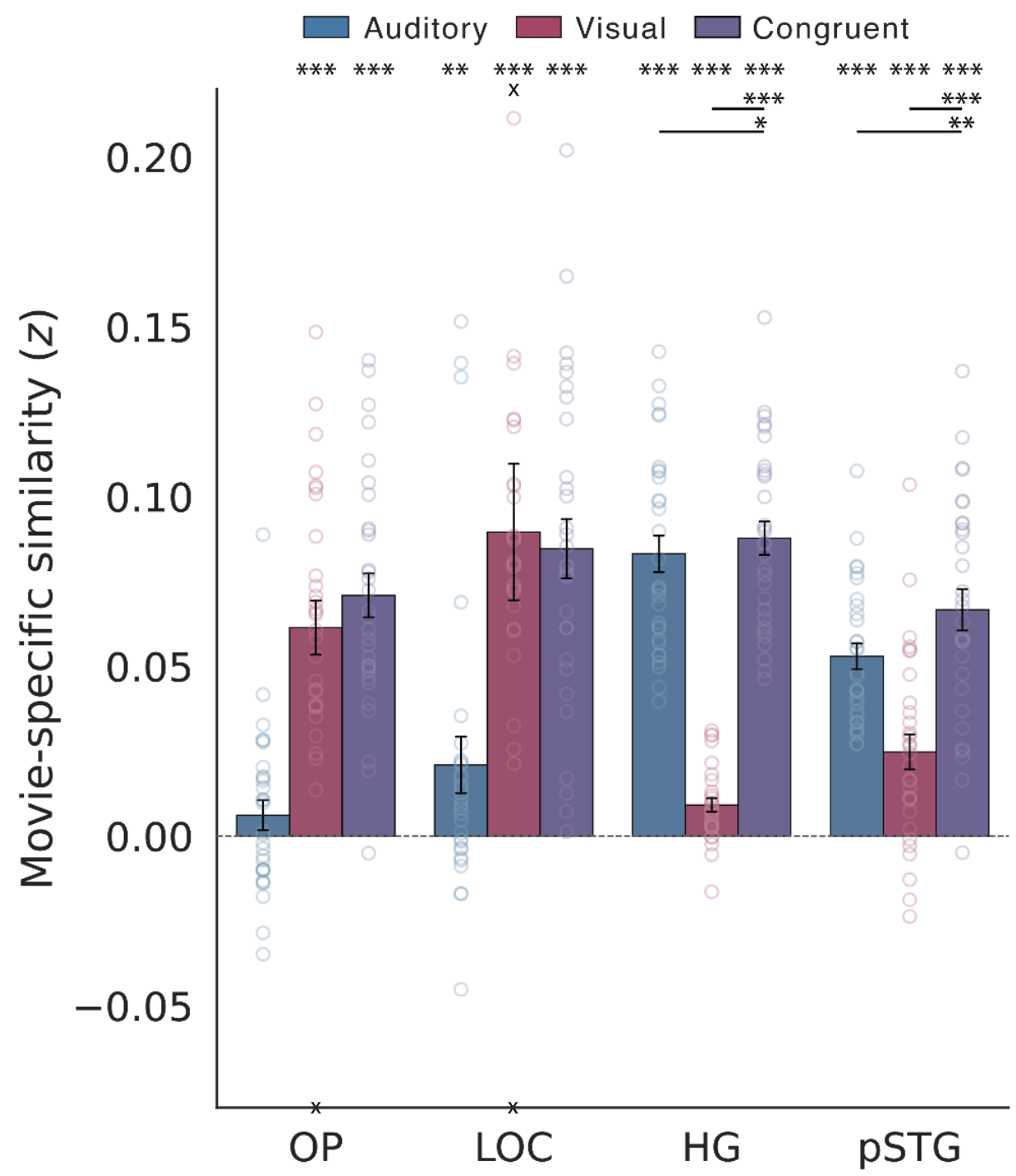

Multivariate RSA results in sensory ROIs. Movie-specific pattern similarity (MSPS) for each condition in sensory cortical ROIs (OP, LOC, HG, pSTG). HG and pSTG showed significant multisensory facilitation. Individual participant data shown as circles; error bars indicate SEM. × markers indicate individual data points outside the plotted y-axis range. * $P < 0.05$, ** $P < 0.01$, *** $P < 0.001$.

These multivariate effects were notably weaker than the patterns for the presented modality. Even so, they suggest some degree of reinstatement of absent modalities. We now report these univariate and multivariate cortical ROI results in the supplement (Figures S2 and S3) and discuss them in relation to retrieval-based accounts of our findings (p. 11), extending the discussion of the split-half analysis above:

“Consistent with this possibility, we observed evidence of perceptual reinstatement in both modalities: auditory-only MSPS was significant in visual cortical ROIs, and visual-only MSPS was significant in auditory cortical ROIs (Figure S3). These multivariate effects were notably weaker than the patterns for the presented modality. Even so, they suggest some degree of reinstatement of absent modalities. Future work that directly examines crossmodal learning will be necessary to fully adjudicate between perceptual- and retrieval-based accounts.”

5. Second, salience. Could it be that the audio-only and video-only stimuli engage external attention to different degrees, or might one condition be lower in perceptual salience than the other? Relatedly, because participants performed a cover task in which they pressed a button when the stimulus ended, the task may place different attentional-switching demands on the two conditions. For example, if the auditory stimuli are relatively lower in perceptual salience, externally orienting to the task may constitute a larger shift for that condition. Such a possibility could plausibly matter for hippocampal responses, given recent evidence that the hippocampal long axis is functionally differentiated, with posterior hippocampal regions showing stronger links to salience- and target-detection-related processing, whereas anterior regions are more closely associated with networks involved in memory representation (Angeli et al., 2025). Consistent with this concern, the whole-brain analysis appears to show large-scale neocortical differences between the auditory-only and video-only conditions, raising the possibility that these conditions engage different large-scale networks in addition to differing in sensory modality alone. One possible way to address this would be to ask whether, across the 6 s movie period, there are greater univariate responses in dorsal attention or salience-related regions for one condition versus the other, which would help clarify whether the contrast reflects differences in external orienting demands rather than sensory modality per se. A second suggestion is to test whether the button-press reaction times for the two conditions in the cover task are matched.

We agree that salience is an important factor to consider when interpreting our findings. As suggested, we compared reaction times in the cover task between the visual-only and auditory-only conditions. We found no significant difference ($t(29) = 1.29$, $P = 0.21$; paired t-test), consistent with the two unisensory conditions being relatively matched in terms of overt task engagement.

Nevertheless, it is possible that perceptual salience could help explain the visually dominant hippocampal responses we observed in our univariate analysis (Figure 2A-B). To address this, we extracted univariate GLM estimates from the salience network (Yeo atlas) for each unisensory condition. Contrary to what might be expected based on a salience-based account of our univariate hippocampal results, we observed greater salience network activity for the auditory-only than the visual-only condition ($M = -0.068$, $P < 0.001$, $SEM = 0.015$). This could be interpreted as inconsistent with a perceptual salience account of greater visual responses in hippocampus ROIs. That said, this interpretation requires reverse inference (assuming that activity in the salience network is a measure of salience) and so we have decided not to include this analysis in the revised manuscript itself.

Importantly, our conclusion is further supported by the finding that incongruent audiovisual conditions elicited lower responses than either unisensory condition across hippocampal ROIs (Figure 2A-B). If low-level salience in either modality were driving these responses, adding a second discordant modality should enhance them. Instead, we observed suppression.

It is important to note that salience might be most relevant to consider for the univariate analyses. Our multivariate effects are defined by movie-specific information content, which subtracts out pattern similarity across different movies. This measure isolates item-level representations from overall response magnitude and is inherently robust to condition-level shifts in salience or attention.

As suggested, we have now added a discussion paragraph about salience (p. 10):

"Auditory and visual stimuli may differ in perceptual salience or attentional demands, complicating the comparison between unisensory conditions. Several aspects of our data argue against a salience-driven account. First, response times in the offset detection behavioral task did not differ between unisensory conditions, indicating relatively matched task engagement ($P > 0.05$). Second, the incongruent audiovisual condition elicited lower univariate responses than both unisensory conditions across hippocampal ROIs (Figure 2A–B); if low-level salience in either modality were driving these responses, adding a second discordant modality should have enhanced them. Third, although salience could in principle modulate the overall magnitude of BOLD responses, our central effects are defined by movie-specific pattern similarity, which controls for the pattern similarity across different movies; as such, our multivariate analyses are inherently robust to condition-level differences in salience or attention."

Minor:

1. Interesting results from cortical MTL structures (parahippocampal cortex, perirhinal cortex, and entorhinal cortex) have been put into the SI, but a full paragraph is given to these results in the Discussion. I'd suggest that authors incorporate these results into the main Results.

We thank the Reviewer for this suggestion. We gave it careful consideration but decided to retain the cortical MTL results in the supplement, given that we needed to add another main figure for the revision and that we wanted to preserve a focus on the substantial set of results from the hippocampus. We continue to engage with the cortical MTL results in the Discussion, where they provide valuable context for interpreting the hippocampal effects. That said, if the Reviewer feels strongly, we'd be happy to move

the cortical MTL results to the main text or to move the cortical MTL discussion to the supplement.

2. It seems surprising that there was not a significant univariate response in the auditory condition in any of the hippocampal areas and subfields examined, given that the authors cite work that demonstrate hippocampal responsiveness to a variety of auditory-only tasks. Including some discussion on the lack of univariate auditory-only response, and if that was expected or unexpected would be helpful.

We thank the Reviewer for raising this important point. Indeed, substantial evidence motivates the hypothesis of a univariate hippocampal response to auditory stimuli, primarily in nonhuman animals (Billig et al., 2022). A null univariate effect is difficult to interpret and may reflect methodological factors (e.g., task design, the modest amplitude of evoked hippocampal BOLD, or acoustic scanner noise). Nevertheless, we believe the analytical dissociation we observed between univariate and multivariate analyses has important theoretical implications that extend beyond the greater sensitivity afforded by the multivariate analysis.

Whereas the univariate GLM contrast reflects overall regional engagement, our multivariate analysis specifically indexes stimulus-specific information carried in distributed voxel-wise patterns. Such patterns are often undetectable in the mean univariate signal, and their presence is consistent with the sparse, distributed coding characteristic of the human hippocampus (Chadwick et al., 2010; Wixted et al., 2014). Furthermore, these multivariate patterns capture movie-specific representations of event- or item-level content, in line with the role of the hippocampus in the rapid encoding of individuated events (Ison et al., 2015). It is important to note that these results do not preclude a univariate response to auditory stimulation. Univariate auditory responses may be detectable in other paradigms that drive higher-SNR hippocampal responses; for example, tasks that impose explicit memory demands or do not rely on stimulus repetitions.

We have added the following text to better clarify our interpretation (p. 10):

“This suggests that auditory content can be encoded in distributed voxel-wise patterns and not readily captured in the mean activity across these voxels, underscoring the need for multivariate approaches to detect non-visual representations in the human hippocampus.

This dissociation may be theoretically informative, as opposed to simply reflecting the greater sensitivity of multivariate methods. Whereas the univariate analysis reflects overall regional engagement, our multivariate RSA indexes stimulus-specific information

carried across distributed voxel patterns. The movie-specific (event-level) structure captured in this analysis is consistent with prominent accounts implicating the hippocampus in the rapid, sparse coding of individuated events (Wixted et al., 2014; Chadwick et al., 2010; Ison et al., 2015). Importantly, this does not preclude a univariate hippocampal response to auditory stimuli, which would be expected given extensive evidence for auditory processing in the hippocampus, primarily observed in non-human animals (Billig et al., 2022). Univariate auditory responses may be identified under conditions that more strongly drive hippocampal activity, such as paradigms imposing explicit memory demands or those not relying on stimulus repetitions."

3. Given how the results focus on a dissociation along the long axis of the hippocampus, it'd be valuable to have more discussion of alternative long axis models beyond the coarse to fine gradient, such as anterior encoding vs posterior retrieval (could congruent presentations be more strongly promoting recall?) or spatial versus non-spatial representations (e.g., Poppenk, 2013)

We thank the Reviewer for this comment. We have added a subsection to the Discussion (*Implications for hippocampal long-axis specialization and lateralization*) that weighs alternative accounts of long-axis specialization against our findings (p. 11-12):

"Our results additionally revealed hemispheric asymmetries along the long axis: multisensory facilitation was strongest in the left posterior hippocampus, whereas crossmodal transfer was driven by the right anterior hippocampus. The human hippocampus displays strong lateralization across a range of memory and navigation tasks (Burgess et al., 2002; Howard et al., 2014; Maguire et al., 1998; Maguire & Frith, 2003; O'Keefe et al., 1998; Spiers et al., 2001). Our results are broadly consistent with a left-hemisphere bias for episodic coding of stimulus content and a right-hemisphere bias for coarser, configural representations that are better suited to abstracting across modality-specific detail. The latter aligns with work showing a right hippocampal bias for statistical learning across different types of visual stimuli (Turk-Browne et al., 2009; Turk-Browne et al., 2010). These interpretations are speculative, as the principles governing hippocampal laterality remain poorly understood. Even so, the asymmetries observed here motivate the use of multisensory stimuli to constrain hypotheses regarding lateralization in the human hippocampus."

Reviewer #2

This manuscript presents the results of a neuroimaging study investigating cross-modal representations of audio-visual movies in the human hippocampus. The authors used

standard univariate analyses and representation similarity analyses of fMRI data acquired in different movie conditions to examine auditory, visual, audio-visual (congruent and incongruent) representations of the movies across the subfields and longitudinal axis of the hippocampus. The results of the RSA showed that the hippocampus represents both auditory and visual scenes. The representation of (congruent) audio-visual scenes was greater than unimodal sensory auditory and visual scenes representations in the posterior hippocampus. Interestingly, patterns were similar across auditory and visual modalities in the anterior hippocampus, suggesting that this region represents scenes irrespective of the modality. The results are discussed in the context of a facilitation / generalization gradient along the hippocampal longitudinal axis. This is a very interesting paper that examines an important research question. I listed below some methodological points that should be addressed to strengthen the paper.

We thank the Reviewer for their constructive feedback and encouraging comments on our manuscript.

Major points:

- Representation of overlapping conditions

As the same movies were used across all conditions, the audio-visual (congruent) diagonal similarity values could be entirely explained by unimodal sensory coding (ie pure auditory or visual coding). The authors compared similarity values between conditions to test whether e.g. congruent audio-visual coding was greater than unimodal sensory coding. I recommend testing for the contribution of these overlapping representations with e.g., regression analyses to give the readers an idea of how much variance in eg the multimodal condition is explained by unimodal representations. Importantly, it is expected that when the overlapping unimodal representations are regressed out from the multimodal representation, the similarity would still be greater than zero (showing eg multimodal representation that is not fully explained by unimodal representations).

We thank the Reviewer for this important comment. We agree that the suggested analysis speaks directly to whether the observed audiovisual effects reflect a conjunctive code rather than a summation of the unisensory auditory and visual representations. To address this, we performed a cross-validated multiple regression analysis, modeling the pattern similarity matrix for the congruent audiovisual condition as a function of the auditory- and visual-only matrices. For each participant and ROI, we fit a regression model over the 10 x 10 Fisher z-transformed similarity matrices (C = congruent audiovisual; A = auditory only; V = visual only):

$$\mathrm{vec}(C) = \beta_0 + \beta_A \cdot \mathrm{vec}(A) + \beta_V \cdot \mathrm{vec}(V) + \varepsilon$$

We estimated the model using leave-one-run-out cross-validation, consistent with our main RSA. On each fold, coefficients were estimated from the training runs and applied to the held-out run, and the resulting estimates were averaged across folds when deriving: (1) the beta coefficients for the auditory- and visual-only conditions; (2) the variance in the audiovisual matrix explained by the unisensory matrices; and (3) the movie-specific pattern similarity (diagonal minus off-diagonal) in the residual audiovisual matrix after regressing out the unisensory contributions.

We evaluated significance using non-parametric sign-flipping randomization (10,000 iterations per ROI) with FDR correction applied across anatomical subdivisions. In addition to hippocampal ROIs, we included two visual and two auditory cortical ROIs for comparison (Harvard-Oxford atlas): the occipital pole (OP) and lateral occipital cortex (LOC) in visual cortex, and Heschl's gyrus (HG) and the posterior superior temporal gyrus (pSTG) in auditory cortex.

The regression coefficients showed expected patterns in visual and auditory cortices: the auditory matrix dominated the prediction of the audiovisual matrix in auditory ROIs, whereas the visual matrix dominated in visual ROIs. In the hippocampus, the audiovisual matrix was best predicted by the visual matrix. As expected, since this analysis modeled the full similarity matrix rather than isolating movie-specific structure, this visual dominance mirrors our univariate GLM findings. These results are shown below for cortical (left) and hippocampal (right) ROIs:

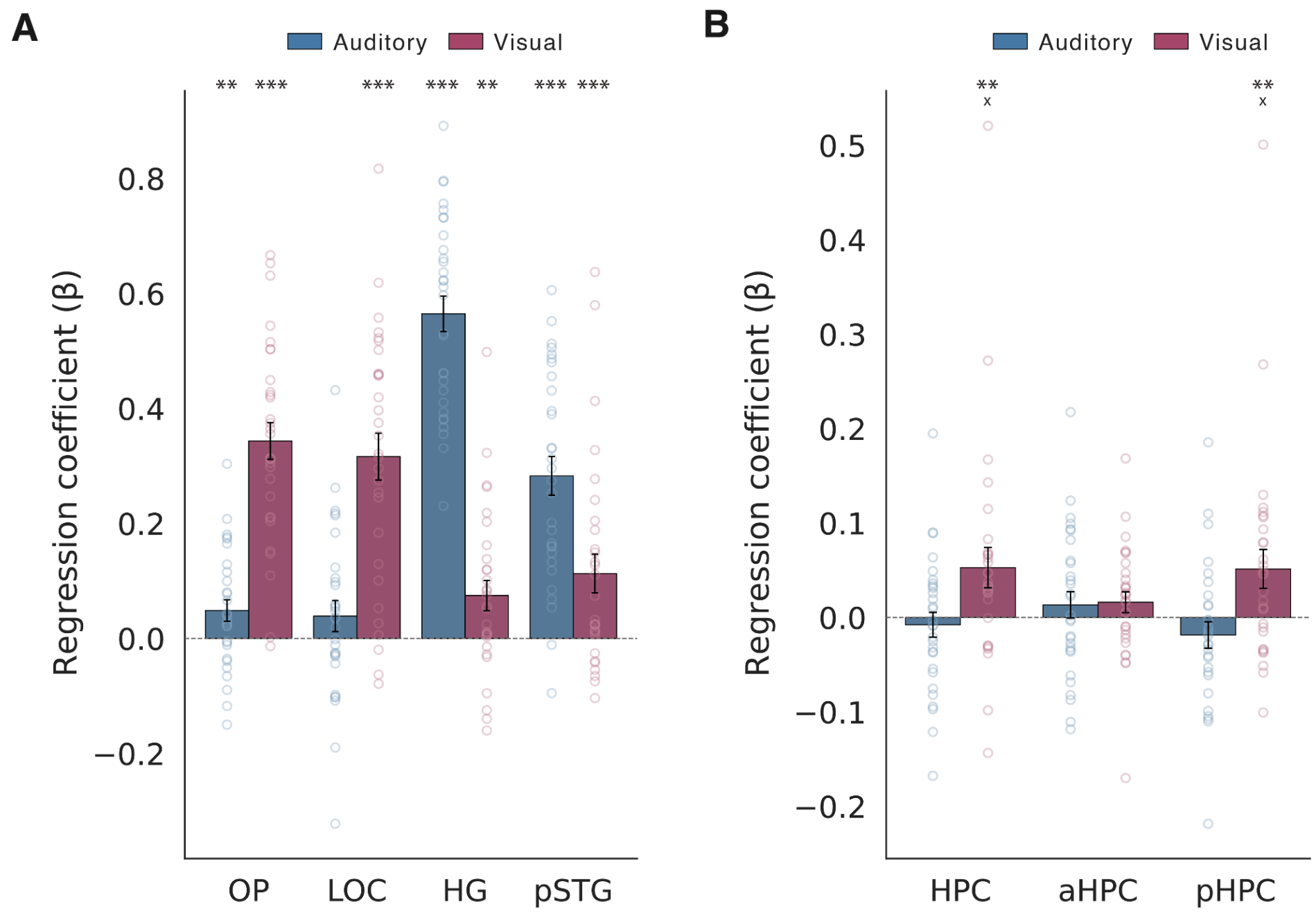

Regression coefficients for auditory and visual predictors. (left) Auditory and visual regression coefficients from the cross-validated multiple regression in sensory cortical ROIs (OP, LOC, HG, pSTG). (right) regression coefficients are shown for the whole hippocampus and anterior/posterior subdivisions. Individual participant data shown as circles; error bars indicate SEM. × markers indicate individual data points outside the plotted y-axis range. * $P < 0.05$, ** $P < 0.01$, *** $P < 0.001$

In sensory cortices, the auditory and visual matrices jointly explained a substantial portion of the variance in the audiovisual matrix. In contrast, across hippocampal ROIs, the unisensory matrices explained no detectable variance. See results below:

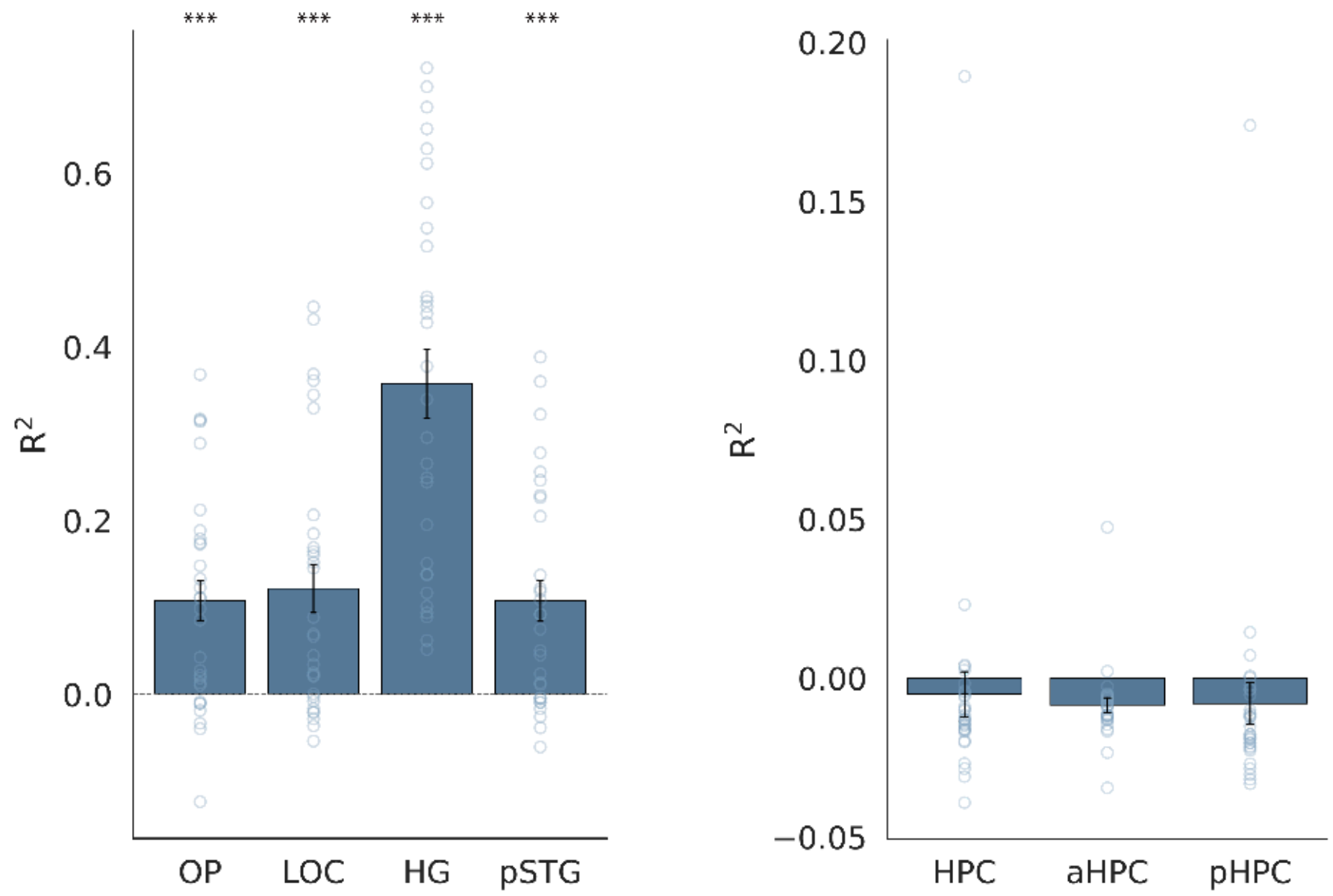

Variance explained in the congruent audiovisual matrix. (left) Variance explained (R^2) by the unisensory matrices in sensory cortical ROIs (OP, LOC, HG, pSTG). **(right)** Variance explained (R^2) by the unisensory matrices in the whole hippocampus and anterior/posterior subdivisions. Individual participant data shown as circles; error bars indicate SEM. *** $P < 0.01$

Finally, after regressing out the unisensory conditions, the residual congruent audiovisual MSPS remained significantly above chance in both sensory and hippocampal ROIs:

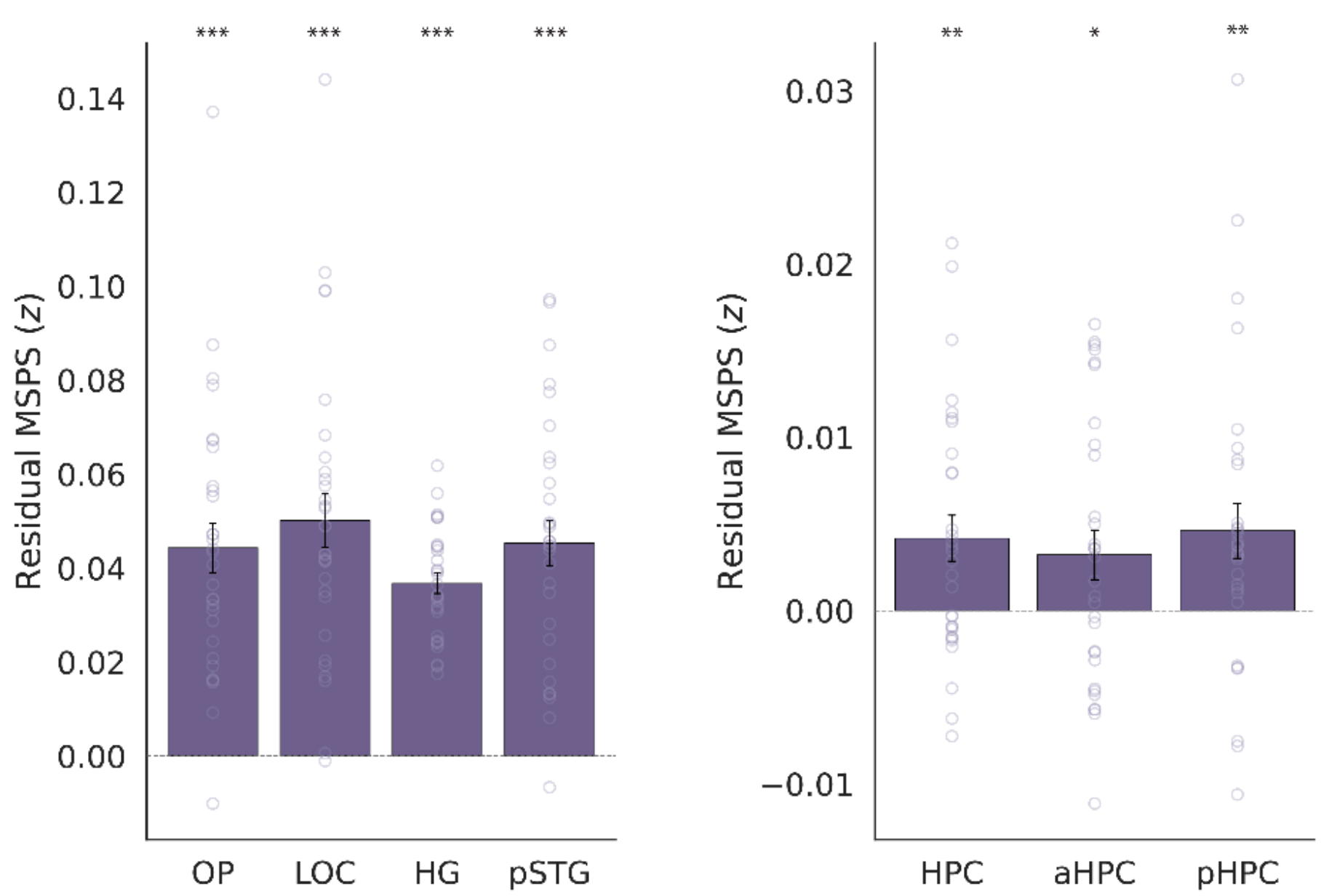

Residual congruent movie-specific pattern similarity. (left) Residual congruent audiovisual MSPS after regressing out the auditory-only and visual-only contributions shown in sensory cortical ROIs (OP,

LOC, HG, pSTG). **(right)** Residual congruent audiovisual MSPS in the whole hippocampus and anterior/posterior subdivisions. Individual participant data shown as circles; error bars indicate SEM. * $P < 0.05$, ** $P < 0.01$, *** $P < 0.001$.

Together, these analyses show that the congruent audiovisual patterns in the hippocampus cannot be explained by a linear combination of the unisensory conditions and carry reliable movie-specific structure after unisensory contributions are regressed out (i.e., residual MSPS above zero). As such, these findings provide evidence consistent with conjunctive audiovisual coding in the human hippocampus.

We present the results from the hippocampal ROIs in the main text (Figure 4) and from the sensory ROIs in the supplement (Figure S5). The newly added Figure 4 additionally includes a schematic to illustrate this approach:

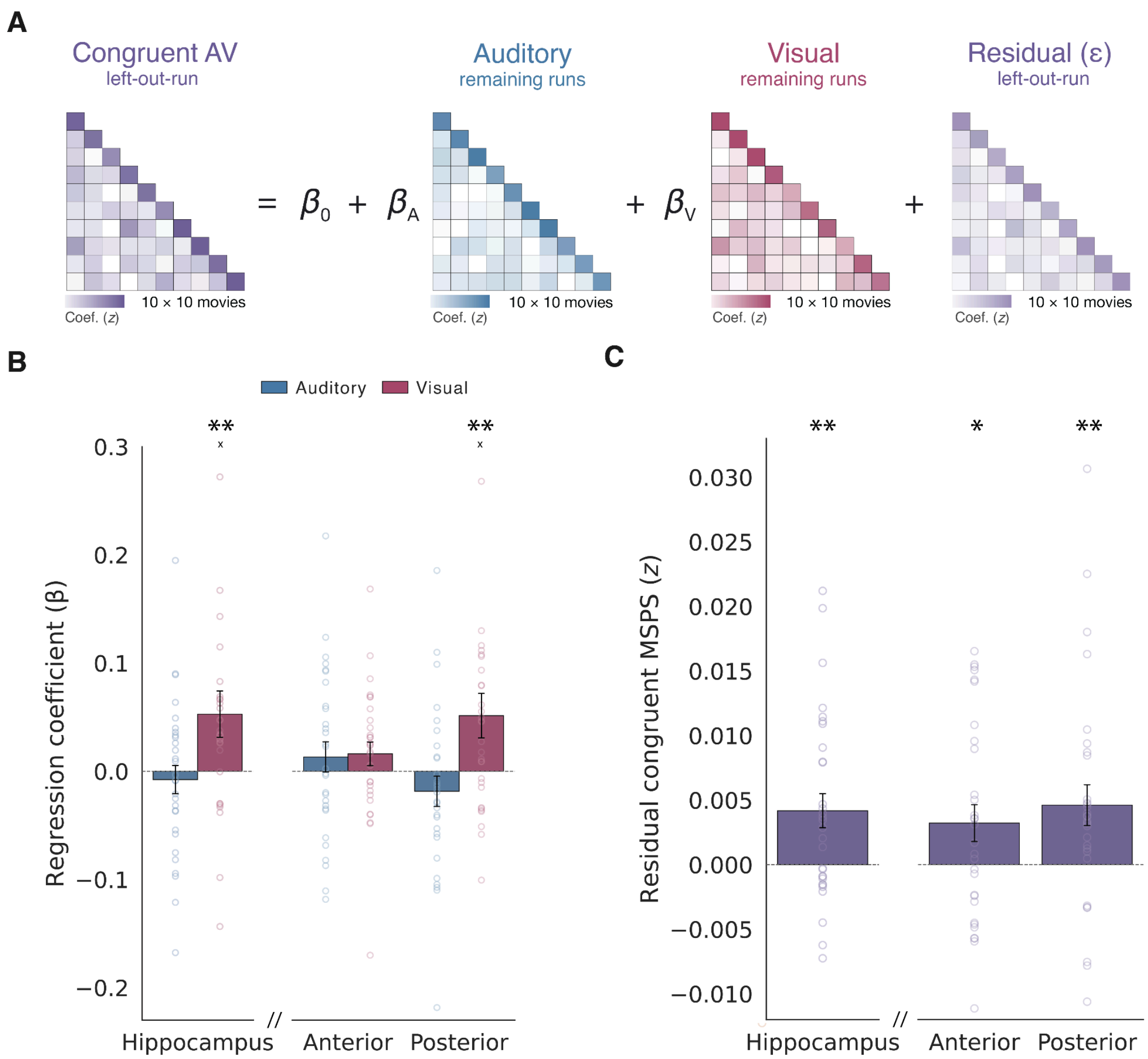

Figure 4: **Conjunctive audiovisual coding in the hippocampus. (A)** To test whether congruent audiovisual representations could be explained by a linear combination of unisensory representations, we

performed a cross-validated multiple regression modeling the congruent audiovisual similarity matrix as a function of the auditory-only and visual-only similarity matrices. Regression coefficients (βA, βV) were estimated by iteratively predicting the congruent matrix on a held-out run from the remaining runs, with estimates averaged across folds. **(B)** Auditory and visual regression coefficients are shown for the whole hippocampus and anterior/posterior subdivisions. **(C)** Residual congruent audiovisual MSPS after regressing out the auditory-only and visual-only contributions. Individual participant data shown as circles; error bars indicate SEM. × markers indicate individual data points outside the plotted y-axis range. * $P < 0.05$, ** $P < 0.01$.

We describe the new analysis in the Methods (p. 15):

"To assess whether the congruent audiovisual representations could be explained by a linear combination of unimodal sensory representations, we performed a cross-validated multiple regression in which the congruent audiovisual similarity matrix was modeled as a function of the auditory-only and visual-only similarity matrices. For each participant and ROI, we fit the following regression over the cells of the 10 (movie) by 10 (movie) Fisher z-transformed similarity matrices:

$$\mathrm{vec}(C) = \beta_0 + \beta_A\,\mathrm{vec}(A) + \beta_V\,\mathrm{vec}(V) + \varepsilon$$

where A, V, and C denote the auditory-only, visual-only, and congruent audiovisual conditions, respectively, and vec(·) denotes vectorization of a 10 × 10 similarity matrix. Model estimation followed the same leave-one-run-out cross-validation procedure as our main RSA. On each fold, regression coefficients were estimated from the cells of the eight training folds and applied to the held-out fold to compute predicted values and residuals. Per-fold residual matrices were averaged across folds to yield a single cross-validated residual matrix per participant and ROI. Cells that could not be computed for a given fold due to trial exclusions remained missing and were dropped from both estimation and evaluation.

From this model we derived three quantities: (1) the regression coefficients β_A and β_V, indexing the contribution of each unimodal condition to the congruent audiovisual similarity; (2) the cross-validated R^2 quantifying the proportion of variance in the congruent audiovisual matrix explained by the unimodal matrices on held-out folds; and (3) the residual congruent audiovisual MSPS, computed as the mean diagonal minus mean off-diagonal of the cross-validated residual matrix, indexing movie-specific pattern similarity in the congruent audiovisual condition that was not accounted for by the unimodal conditions."

We further report these findings in the Results section (p. 6):

“Critically, congruent audiovisual MSPS could reflect a conjunctive audiovisual code, in which congruent stimuli evoke movie-specific structure not present in either modality alone, or simply a linear combination of the unisensory representations. To test this, we ran a cross-validated multiple regression, modeling the congruent audiovisual similarity matrix as a function of the auditory-only and visual-only similarity matrices (Figure 4A). Specifically, we estimated these coefficients by predicting each held-out congruent run from the remaining unisensory runs, following our main RSA approach (see Methods). Averaging across folds, we derived the auditory and visual regression coefficients, the variance explained (R^2), and the residual congruent audiovisual MSPS. The visual-only coefficient was significant in the whole hippocampus ($P = 0.003$) and posterior hippocampus ($P = 0.005$; Figure 4B), whereas the auditory-only coefficient was not significant in any hippocampal ROI (all $P > 0.05$). This visual dominance was expected, as the regression models the full similarity matrix rather than isolating movie-specific structure. Critically, the unisensory matrices explained no significant variance in any hippocampal subdivision (all $P > 0.05$). This was in stark contrast to visual and auditory sensory ROIs in cortex, which accounted for substantial variance ($R^2 \approx$ 11–36%; for full results in sensory ROIs, see Figure S5). Finally, the residual congruent audiovisual MSPS remained above baseline in the whole hippocampus ($P = 0.002$), anterior hippocampus ($P = 0.019$), and posterior hippocampus ($P = 0.005$; Figure 4C). Together, these findings indicate that the hippocampus carries movie-specific audiovisual structure that is not reducible to its unisensory components. For full statistical reporting, see Table S5.”

And discuss its implications in the revised Discussion (p. 11):

“Importantly, these congruent audiovisual representations were not reducible to their unisensory components. Using a cross-validated multiple regression, we found that movie-specific pattern similarity for congruent stimuli remained reliably above zero after regressing out the auditory-only and visual-only similarity matrices. This indicates a conjunctive audiovisual code in the hippocampus, in which congruent stimuli elicited unique movie-specific structure. This contrasts with sensory cortical ROIs, in which the unisensory matrices accounted for substantial variance in the congruent representation. Nevertheless, residual conjunctive structure was evident in both sensory areas and the hippocampus.”

- Additional "control" ROIs

I recommend including additional "unimodal sensory" ROIs that would serve as control ROIs to test for the specific of "primary" sensory coding and e.g. test for visual coding in both a visual and an auditory ROIs, and for auditory coding in both auditory and visual ROIs (and potential facilitation / generalization in these ROIs as well). I also suggest

adding "integration" ROIs and test unimodal, multimodal sensory and cross-modal coding in these cortical regions as well. Together with the regressions mentioned above, these additional analyses will allow for a better characterization of the specificity of the results reported in the manuscript. Note that the point above might require moving away from RSA approaches as Pearson correlation can be an unreliable measure of pattern similarity when there are differences in mean univariate activation between conditions (that you could expect between unimodal regressors in these specific ROIs, see Walther et al., 2016). Also note that the whole brain search light analysis partially addresses this comment (but see point above on potential confound due to differences in univariate activation for unimodal conditions). But it is important to show similarity values for the different conditions (compare them between conditions and provide the regressions mentioned above) to test for the specificity of the hippocampal results.

As shown in our previous response, the cortical ROIs displayed a distinctive profile in which the auditory and visual unisensory conditions accounted for a substantial proportion of the variance in the audiovisual matrix. To fully address this comment, we also ran our univariate GLM and main RSA in these sensory ROIs, which included the pSTG as a canonical audiovisual integration region. Each modality elicited clear univariate responses and MSPS in respective sensory cortices. The pSTG additionally showed robust multisensory facilitation in the multivariate analysis. The absence of a corresponding univariate facilitation likely reflects the spatially localized nature of this effect, as portions of the pSTG demonstrate facilitation in our whole-brain analysis (Figure 2D). These results are now included as a supplementary figure (Figure S3):

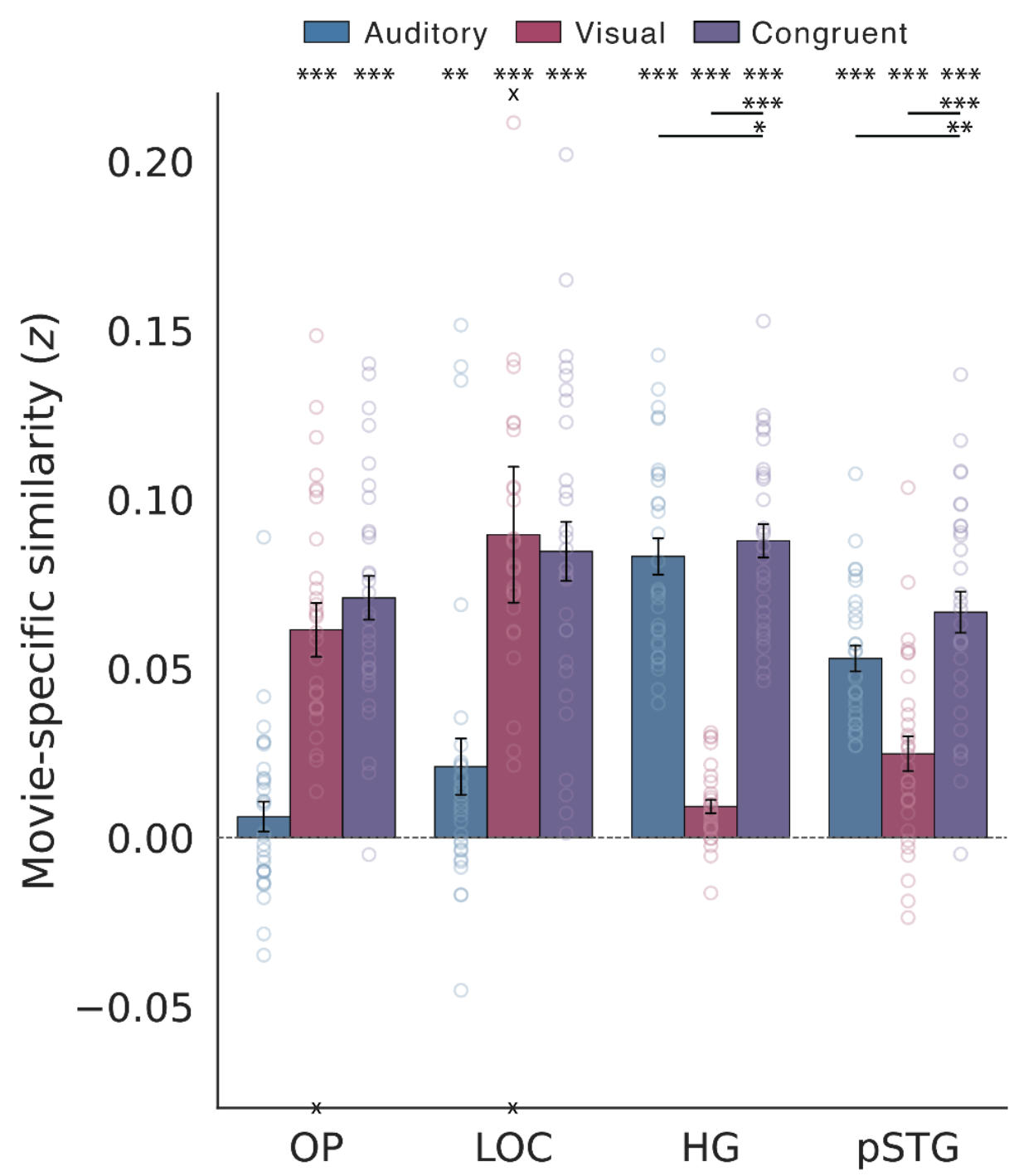

Multivariate RSA results in sensory ROIs. Movie-specific pattern similarity (MSPS) for each condition in sensory cortical ROIs (OP, LOC, HG, pSTG). HG and pSTG showed significant multisensory facilitation. Individual participant data shown as circles; error bars indicate SEM. × markers indicate individual data points outside the plotted y-axis range. * $P < 0.05$, ** $P < 0.01$, *** $P < 0.001$.

- Incongruent condition

Please also report the similarity values for the incongruent condition as this would provide a good "surrogate" analysis in which similarity values should be close to zero between diagonal and off-diagonal.

We thank the Reviewer for this comment. Notably, we do not expect incongruent MSPS to be near zero. As we note in the paper, this condition cannot be compared in a straightforward manner to the congruent condition (as was possible in our univariate analyses) because each run contained a unique set of incongruent audiovisual pairings (e.g., ambulance video was paired with skateboarding audio in run 1 and with typewriter audio in run 2). However, each modality still carries movie-specific information across runs; for example, an ambulance audio track is always paired with a distinct visual component within each run. This results in “incongruent visual” and “incongruent auditory” matrices, allowing us to test whether incongruence suppresses pattern similarity relative to the unisensory conditions (despite both having stable audio or video).

Comparing the mean of the unisensory and incongruent conditions, we found no significant difference in the hippocampus (Figure S6A–B), but reliable incongruence-driven suppression in the parahippocampal cortex, the posterior STG, and several other cortical regions in our searchlight analysis. We now clarify this logic in the manuscript (p. 6):

“Note that this multivariate analysis could not be performed straightforwardly in the incongruent condition because each run contained a unique audiovisual combination. However, each modality still carries movie-specific information across runs; for example, an ambulance audio track is always paired with a distinct visual component within each run. This results in “incongruent visual” and “incongruent auditory” matrices, allowing us to test whether incongruence suppresses pattern similarity relative to the unisensory conditions (despite both having stable audio or video). Although we did not find any significant differences in the hippocampus (Figure S6A), we observed a significant suppression of MSPS for the incongruent relative to unisensory conditions in the PHC (Figure S6B) and at the whole-brain level (Figure S7).”

We report these results within regions of interest in Figure S6:

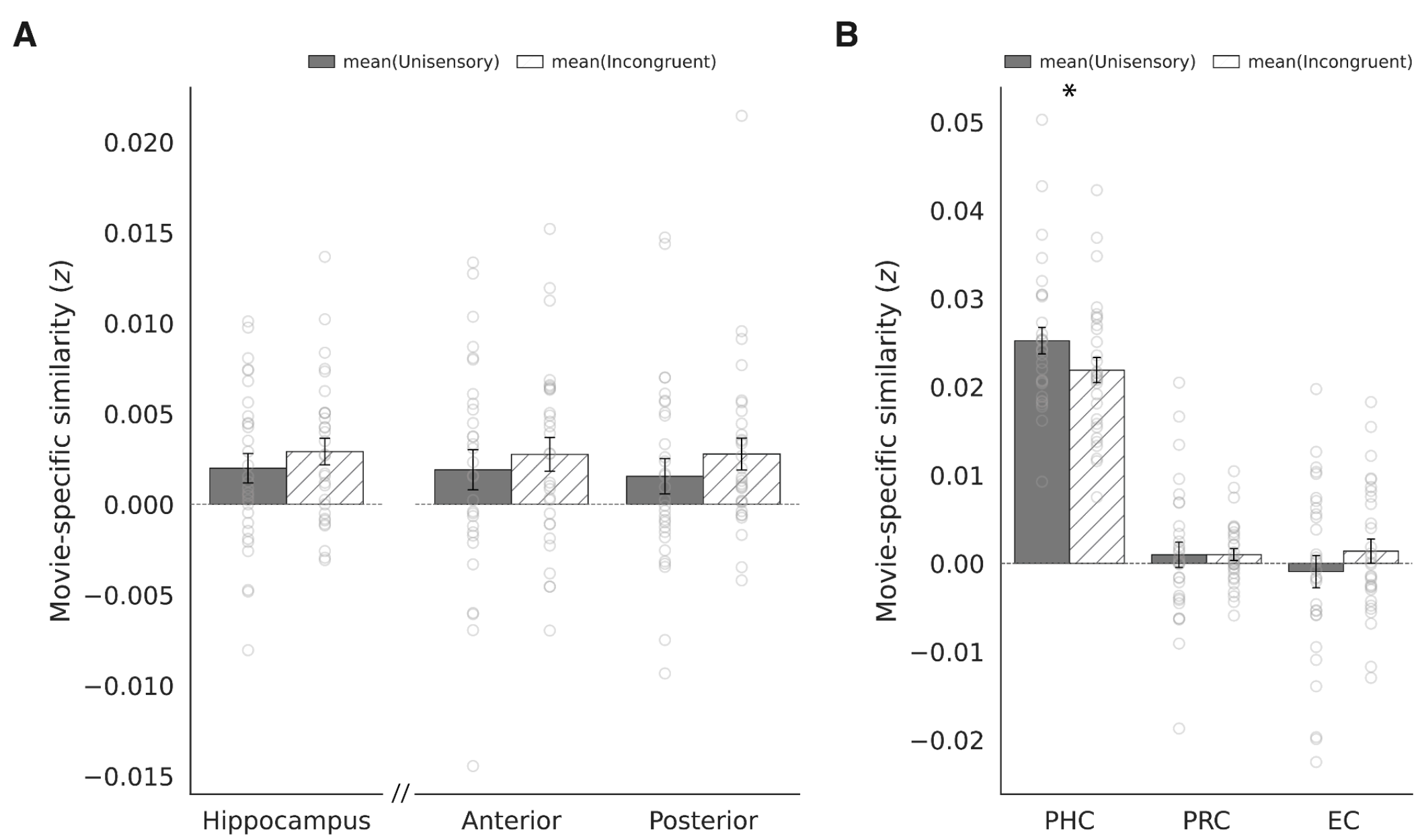

Figure S6: **Unisensory versus incongruent MSPS in the hippocampus and MTL cortex. (A)** No significant differences were observed between incongruent and unisensory (average of auditory and visual) MSPS in the whole hippocampus and anterior/posterior hippocampus **(B)** PHC showed greater MSPS for unisensory relative to incongruent conditions, suggesting a catastrophic interaction between non-matching auditory and visual content. There were no significant differences in PRC or EC. Individual participant data shown as circles; error bars indicate SEM. * $P < 0.05$

And at the whole-brain level in Figure S7:

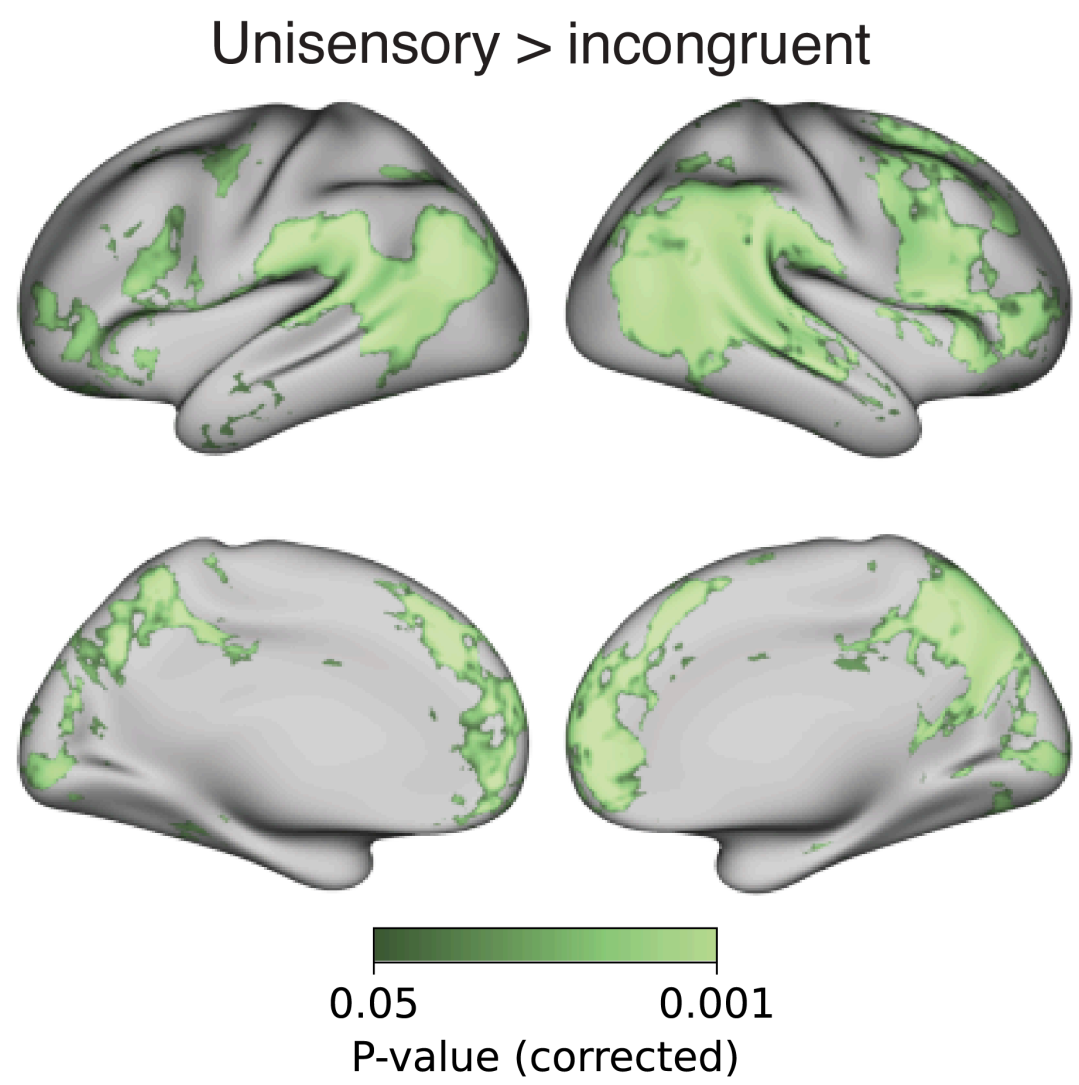

Figure S7: **Whole-brain RSA searchlight of unisensory versus incongruent pattern similarity.** MSPS was significantly reduced for incongruent relative to unisensory (average of auditory and visual) throughout widespread cortical sites, including the posterior STG, lateral occipital cortex, angular gyrus, posteromedial cortex, and the lateral and medial prefrontal cortex. A full list of peak activations is shown in Table S6. Corrected using TFCE at *P* < 0.05.

- Familiarization. As participants were familiarized with the stimuli before the scanning session, one could argue that the unimodal sensory conditions might be contaminated by the other modality (the visual only condition of e.g. "the train movie" might trigger auditory imagery and vice versa). The regression analyses mentioned above might help decipher the contribution of unimodal sensory conditions to the effects that are reported (e.g., does the auditory condition trigger visual coding in a visual ROI). It is also important to reflect on such potential limitations in the discussion.

Thank you for raising this important question (also brought up by Reviewer #1). We addressed how stimulus repetitions influenced our effects in two complementary analyses. As reported in our initial submission, we leveraged our cross-validated RSA, in which each run is held out and correlated with the average of the remaining runs. This yields nine estimates (one per fold), each corresponding to a run's position in the task. A retrieval-based contribution should grow with repeated exposure and learning, and therefore produce larger effects in later held-out runs. We therefore labeled the cross-validation folds by held-out run position (runs 1–9) and correlated this with the magnitude of effects of interest (multisensory facilitation and crossmodal transfer). If repeated exposure strengthened retrieval- or imagery-based contributions, we would expect a positive correlation between run position and effect magnitude. We found no

significant correlation for multisensory facilitation in posterior hippocampus or crossmodal transfer in anterior hippocampus (p. 6):

““The multivariate effects reported above could in principle benefit from memory retrieval, with repeated stimulus exposure allowing for reinstatement of movie-specific details or mental imagery of the absent modality. If so, we would expect that the magnitude of these effects should increase across repeated stimulus presentations. We tested this possibility in two complementary analyses. First, we leveraged the leave-one-run-out cross-validation RSA procedure, in which each run is held out and correlated with the average of the remaining runs. This yields 9 estimates (one per fold), each corresponding to a run’s position in the task. We therefore labeled the cross-validated folds by held-out run position (runs 1–9) and correlated run position with the magnitude of our effects of interest (multisensory facilitation and crossmodal transfer) in that run. If repeated exposure strengthened retrieval- or imagery-based contributions, we would expect a positive correlation between run position and effect magnitude. Neither multisensory facilitation in the posterior hippocampus (congruent audiovisual > visual MSPS; Spearman's $\rho = 0.055$, $P = 0.38$) nor crossmodal transfer in the anterior hippocampus ($\rho = 0.037$, $P = 0.55$) strengthened with more stimulus repetition across runs.”

To further investigate this issue in the revised manuscript, we performed a split-half analysis. Specifically, we computed cross-validated RSA separately within the first four runs and the last four runs (omitting the middle fifth run to balance the comparison). Within each half, we estimated multisensory facilitation in the posterior hippocampus and crossmodal transfer in the anterior hippocampus. We observed a trend toward decreased facilitation in posterior hippocampus and increased crossmodal transfer in anterior hippocampus in the later runs. However, neither effect differed significantly between the two halves (paired randomization test, 10,000 iterations). We report this analysis in the revised manuscript (p. 6-7):

“Second, we ran a split-half analysis comparing effects estimated within the first four runs versus the last four runs (omitting the middle run for balance; Figure S10). In the first half, the posterior hippocampus showed significant MSPS for the auditory-only ($P = 0.017$) and congruent audiovisual ($P = 0.050$) conditions; in the second half, there were no significant effects. In contrast, crossmodal transfer in the anterior hippocampus was significant in the second half ($P = 0.005$) but not the first ($P = 0.12$). However, neither multisensory facilitation nor crossmodal transfer differed significantly between the two halves (both $P > 0.05$). These results raise the possibility that facilitation and transfer might be affected differently by stimulus repetitions, perhaps because crossmodal transfer involves learning amodal abstractions.”

And include the figure in the revised supplement (Figure S10):

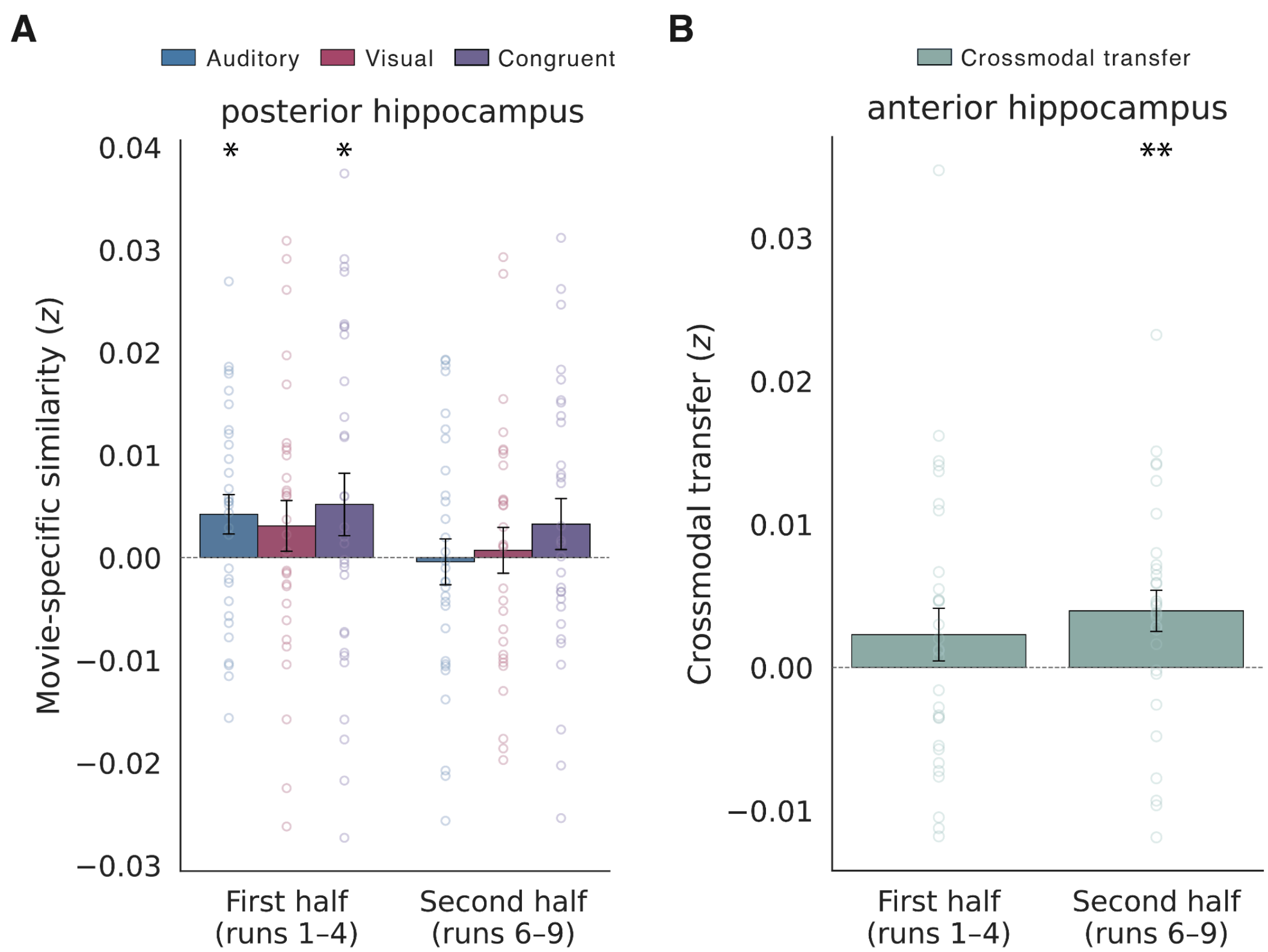

Figure S10: **Split-half analysis of multisensory facilitation and crossmodal transfer. (A)** MSPS for each condition in the posterior hippocampus, estimated separately within the first half (runs 1–4) and second half (runs 6–9) of the experiment. **(B)** Crossmodal transfer MSPS in the anterior hippocampus across the same two halves. Individual participant data shown as circles; error bars indicate SEM. * $P < 0.05$, ** $P < 0.01$.

We additionally included a paragraph in the revised Discussion about the role of retrieval (p. 11):

"Although our analyses across repeated exposures provided limited support for a retrieval-based account of these effects, we observed a trend toward enhanced crossmodal transfer in later runs, which suggests that these effects may be driven in part by learned audiovisual associations (Figure S10). In fact, even without an increase across runs, it is possible that the pre-experimental exposure phase enabled retrieval processes even early in the experiment."

Minor points:

- Performance on the offset detection task: I am not sure I follow what the p values represent here - what is being tested?

"Performance in this offset detection task was high across conditions (auditory: mean accuracy across participants, M = 0.94, standard error of the mean, SEM = 0.006, $P < 0.001$; visual: M = 0.98, SEM = 0.006, $P < 0.001$; congruent: M = 0.98, SEM = 0.003, $P < 0.001$; incongruent: M = 0.99, SEM = 0.003, $P < 0.001$)."

We apologize for the lack of clarity. These p-values reflected whether mean accuracy in each condition exceeded chance, using a one-tailed sign-flipping randomization test against chance level at 50%. On reflection, however, there is no well-defined analytic baseline in our behavioral offset detection task. We have therefore opted to remove these tests against chance from the revised manuscript and instead simply report the mean and SEM of accuracy for each condition.

- What makes a trial "correct"? A response before within the 1s window? Please specify.

A trial was scored as correct when the participant made a button press within the 1-s window following movie offset. Responses made during the movie, or more than 1 s after its offset, were scored as incorrect. We have clarified this in the main text (p. 2):

“A response was scored as correct if it fell within this 1-s post-offset window, whereas presses during the movie or after the window were scored as incorrect”

- Figure 3A: As the RSA is currently only performed on hippo ROI, I suggest altering the ROI depiction.

Thank you for this suggestion. We have updated our RSA schematic accordingly:

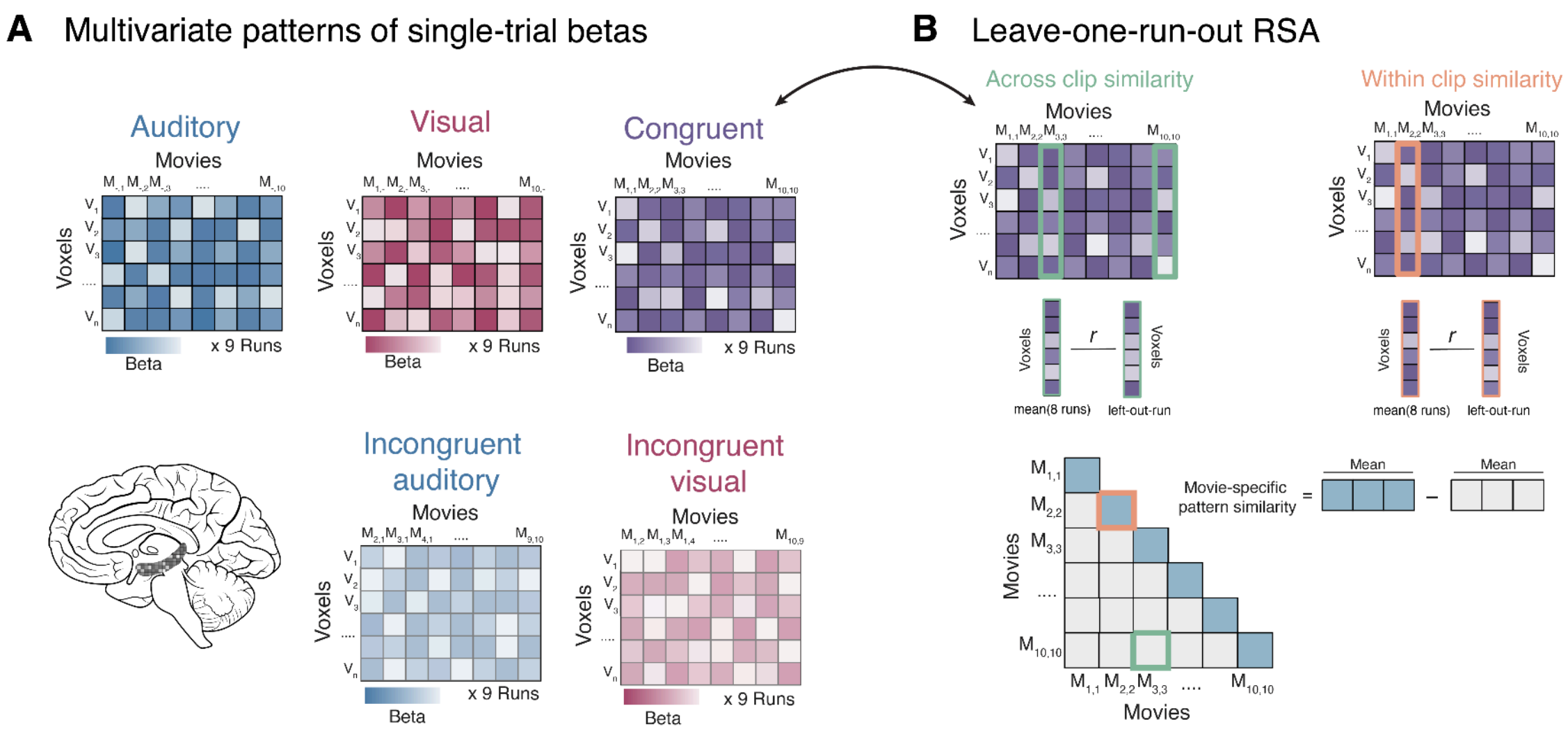

- Connectivity analyses can be performed with such event-related design. Therefore, I am not sure I follow the statement below:
"The current event-related design precludes a rigorous analysis of the interactions across these regions, which could be addressed using longer-duration audiovisual stimuli to enable connectivity analyses."

We agree that connectivity analyses can be performed with an event-related design such as ours. We have removed this statement.

Reviewer #3

This manuscript tackles a fundamental, yet under-explored, question about hippocampal representations regarding dynamic stimuli that vary in their sensory content (visual-only information, auditory-only, coherent audiovisual, incongruent audiovisual). They make the well-taken point that much of the literature (especially within human cognitive neuroscience) has largely focused on visual stimuli (e.g., pictures of objects as used in standard item recognition tasks that proliferate among memory researchers). To investigate the potential broader representational role of MTL, the authors used a clean, well-powered design in which participants viewed (or, in the audio-only condition, listened) to 10 clips in each of the critical conditions, repeated across a 9 runs of fMRI scanning. In the audio, visual, and congruent conditions, the 10 clips remained the same in each run. For the incongruent condition, audio/visual tracks were shuffled such that the 10 clips were a run-unique pairing. This then enabled 1) univariate and 2) multivariate ("movie specific pattern similarity") analyses.

Replicating decades of work, they find univariate HC activity for both visual-only clips and visual + audio (multisensory) clips, irrespective of congruency. Notably, across MTL, the only region to show univariate representations of auditory-only clips was PHC, although this was actually a negative activation (although this is perhaps a bit hard to interpret since I'm assuming they're using the ITI as an implicit baseline). Turning to multivariate representations, they find a quite different pattern of results in which HC seems to demonstrate clip-specific representations for audio-only and congruent audiovisual clips but not visual alone. This perhaps gets at an intriguing distinction between representational vs. mnemonic functions of the HC, although the lack of changes in MSPS across repeated viewings suggests a simple mnemonic explanation may be insufficient.

They additionally find what they frame as a compelling distinction between anterior/postierior HC in which aHC shows elevated within movie similarity regardless of presentation modality (audio-only/visual-only) whereas pHC does not show this "cross-modal transfer." There are additional analysts split by HC subfields, although these do not add substantially. Whole brain findings reveal the "usual" cortical players for unimodal stimuli and results broadly consistent with the nascent but growing naturalistic literature suggesting a role for "posterior medial cortex" in conjunctive coding as well as modality-invariant representation of narratives (Fig 5 C & D).

This paper is well-written with little to no grammatical or linguistic elements that would hinder our understanding. Methods (both stimulus/experimental design) and analytical approach are appropriate. This work follows an emerging trend of asking questions about putative memory structures but without a clear memory component which may present interesting challenges for memory researchers interested in the role of the HC. I did not review the first submission of this paper, but I have the following thoughts on this revision:

We sincerely thank the Reviewer for their careful summary and constructive suggestions.

The major point I find myself wrestling with is the discrepancy between our interpretation of what HC is doing between the univariate and multivariate analyses (considering also prior literature showing in particular HC representations of visual-only items). Do they think the key difference here is about a viewing-only vs. memory task? This is a very interesting theoretical implication of their work and one I felt was under-specified in the discussion.

We thank the Reviewer for raising this important point, which was also raised by Reviewer #1. We fully agree that the dissociation between our univariate and multivariate results carries important theoretical implications that likely extend beyond the greater sensitivity of multivariate methods.

Whereas the univariate GLM contrast reflects overall regional engagement, our multivariate analysis specifically indexes stimulus-specific information carried in distributed voxel-wise patterns. Such patterns are often undetectable in the mean univariate signal, and their presence is consistent with the sparse, distributed coding characteristic of the human hippocampus (Chadwick et al., 2010; Wixted et al., 2014). Furthermore, these multivariate patterns capture movie-specific representations of event- or item-level content, in line with the role of the hippocampus in the rapid encoding of individuated events (Ison et al., 2015). It is important to note that these results do not preclude a univariate response to auditory stimulation. Univariate auditory responses may be detectable in other paradigms that drive higher-SNR hippocampal responses; for example, tasks that impose explicit memory demands or do not rely on stimulus repetitions.

We have added the following text to better clarify our interpretation (p. 10):

“This suggests that auditory content can be encoded in distributed voxel-wise patterns and not readily captured in the mean activity across these voxels, underscoring the need for multivariate approaches to detect non-visual representations in the human hippocampus.

This dissociation may be theoretically informative, as opposed to simply reflecting the greater sensitivity of multivariate methods. Whereas the univariate analysis reflects overall regional engagement, our multivariate RSA indexes stimulus-specific information carried across distributed voxel patterns. The movie-specific (event-level) structure captured in this analysis is consistent with prominent accounts implicating the hippocampus in the rapid, sparse coding of individuated events (Wixted et al., 2014; Chadwick et al., 2010; Ison et al., 2015). Importantly, this does not preclude a univariate hippocampal response to auditory stimuli, which would be expected given extensive evidence for auditory processing in the hippocampus, primarily observed in non-human animals (Billig et al., 2022). Univariate auditory responses may be identified under conditions that more strongly drive hippocampal activity, such as paradigms imposing explicit memory demands or those not relying on stimulus repetitions.”

Given that this paper is framed as a "first foray," it may be helpful to report the extent of cortical regions (eg, Figs 2, 5, & supplementals) in a supplemental table. In the main figs, they could also consider outlining the extent of brain regions so it's clearer to the

reader. Otherwise, it feels a bit like "the naturalistic processing bits of DMN," but I think there may be some important specificity.

We agree with the Reviewer that reporting the extent of cortical regions would be helpful in interpreting our whole-brain results. To address this, we extracted peak activations from clusters in our whole-brain maps, identified using threshold free cluster enhancement (TFCE) at $P < 0.05$. We then assigned anatomical labels for each peak using the Harvard-Oxford atlas. We now report these anatomical delineations, along with the extent of each cluster, in Tables S2 and S6. These include peak activation for all contrasts reported in the main text and supplementary figures.

These annotations revealed important regional specificity not explicitly reported in our initial submission, including facilitation effects in the insula, temporal pole, as well as subcortical structures previously implicated in multisensory processing. Furthermore, we show that crossmodal transfer effects are localized to specific sensory ROIs as well as the middle frontal gyrus, consistent with work into cortical amodal representation across audition and vision. Given that the whole-brain maps covered widespread regions, we identify regions with peak activation in the main text rather than delineating boundaries directly on the whole-brain maps.

This includes greater specification in univariate GLM maps (p. 5): "To assess multisensory facilitation, we performed a conjunctive analysis of voxels in which activation for the congruent audiovisual condition significantly exceeded both of the unisensory auditory and visual conditions. This contrast revealed activity in several regions, including bilateral parietal operculum, insula, angular gyrus, temporal pole, and subcortical activity in the thalamus (Figure 2D). Finally, a contrast between congruent and incongruent audiovisual conditions revealed greater activation in bilateral medial prefrontal cortex for congruent and in left lateral prefrontal cortex for incongruent (Figure 2E). Together, these whole-brain effects validate the paradigm by replicating established patterns of unisensory and multisensory processing in the neocortex. The full list of peak coordinates for these contrasts is provided in Table S2."

Additionally in describing the whole-brain RSA searchlight results (p. 8): "Next, we performed a conjunctive contrast to identify regions in which congruent audiovisual stimuli exceeded both unisensory conditions. This revealed multisensory facilitation across the superior temporal lobe, including posterior STG and adjacent posterior perisylvian cortex (planum temporale and parietal operculum), as well as in the insula, temporal pole, and amygdala (Figure 6C). Crossmodal transfer effects at the whole-brain level were robust across perisylvian cortex, angular gyrus, precuneus, and

lateral visual cortex (Figure 6D). The full list of peak coordinates for these contrasts is provided in Table S6."

And in the caption of the unisensory versus incongruent searchlight figure in the supplement (Figure S7): "Whole-brain RSA searchlight of unisensory versus incongruent pattern similarity. MSPS was significantly reduced for incongruent relative to unisensory (average of auditory and visual) throughout widespread cortical sites, including the posterior STG, lateral occipital cortex, angular gyrus, posteromedial cortex, and the lateral and medial prefrontal cortex. A full list of peak coordinates is provided in Table S6."

To further specify cortical responses in the revised manuscript, we ran our univariate and multivariate analyses in four additional cortical ROIs spanning both modalities and both lower- and higher-level processing: occipital pole (OP) and lateral occipital cortex (LOC) for visual cortex; Heschl's gyrus (HG) and the posterior superior temporal gyrus (pSTG) for auditory cortex. The pSTG was additionally selected as a well-characterized audiovisual integration region. We found expected univariate and multivariate patterns in visual and auditory ROIs. In pSTG, we additionally find multisensory facilitation in line with a broad literature on audiovisual integration in the human brain. We show these results for our univariate analysis in Figure S2:

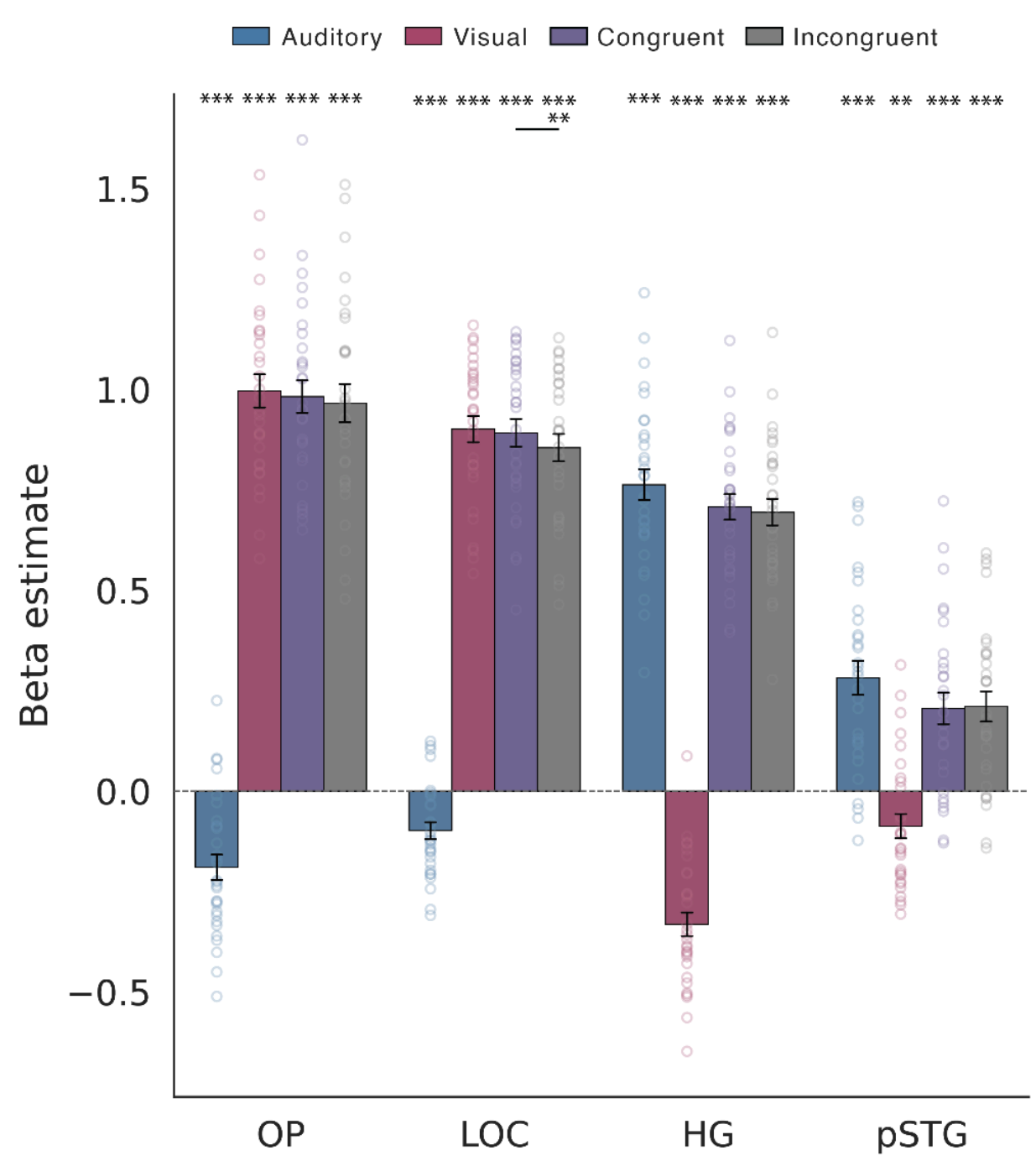

Univariate GLM results in sensory ROIs. Mean beta estimates for each condition in sensory cortical ROIs (OP, LOC, HG, pSTG). Individual participant data shown as circles; error bars indicate SEM. ** *P* < 0.01, *** *P* < 0.001.

And for multivariate analysis in Figure S3:

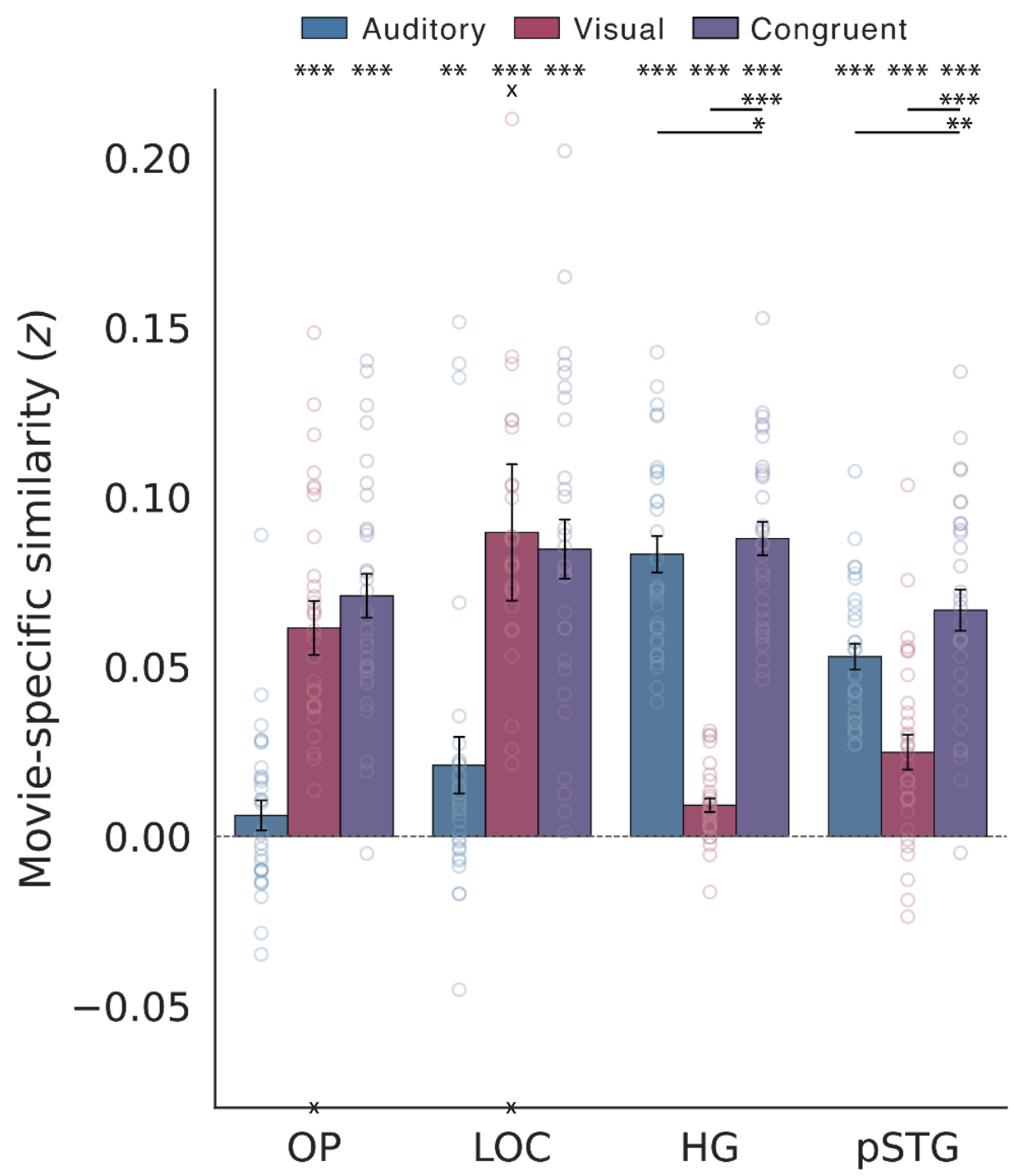

Multivariate RSA results in sensory ROIs. Movie-specific pattern similarity (MSPS) for each condition in sensory cortical ROIs (OP, LOC, HG, pSTG). HG and pSTG showed significant multisensory facilitation. Individual participant data shown as circles; error bars indicate SEM. × markers indicate individual data points outside the plotted y-axis range. ** *P* < 0.01, *** *P* < 0.001.

Again, perhaps in part because the authors frame the current work as a first pass in an under-explored domain, but, in addition to reporting whole HC and results split by long axis, they additionally report results by HC subfield. To me, there are several problems here: (1) they do not give any theoretical motivation (nor can I see a compelling one) WHY you'd expect differences amongst subfields in this regard. I actually wonder if instead the subfield findings should be in the supplemental. And 2) given their subfield results, it is sometimes challenging to reason about how their long axis findings could arise. For instance, in Fig 4, given that much of aHC is comprised of CA1 finding MSPS that is indistinguishable from zero, how can it be that aHC shows a strong MSPS effect in panel B?

We thank the Reviewer for this comment. We had two motivations for examining hippocampal subfields. First, given their differential roles in pattern completion and separation, we reasoned that multisensory representations might dissociate across subfields. Second, recent work in the macaque hippocampus identified distinct proportions of cells responding to congruent versus incongruent face-voice pairings across subfields (Tyree et al., 2023). That said, we agree that the subfield results do not contribute substantively to the interpretation of our main findings. We have therefore moved them to the supplement as suggested (Figures S1) and clarified this motivation in the main text (p. 5):

"We focused ROI analyses on the whole hippocampus, anterior and posterior hippocampus, the left and right hippocampus, and hippocampal subfields CA1, CA2/3, DG, and subiculum. We examined subfields given evidence for differential audiovisual selectivity across subfields in the primate hippocampus (Tyree et al., 2023)."

Regarding the tension between the CA1 and anterior hippocampus results, the anterior hippocampus comprises some portion of all subfields, so crossmodal transfer there need not be carried by CA1 and may instead be concentrated in anterior portions of other subfields. As shown in the next comment, the new laterality analyses suggest that these effects may be organized along the long axis and across hemispheres rather than localized to individual subfields.

Moving beyond subfields, I would have liked to see results either split by hemisphere or, better yet, hemisphere statistically compared. HC exhibits other laterality effects and so it could help provide a helpful baseline expectation for future work in this domain.

We thank the Reviewer for this suggestion, which revealed an interesting set of results. We split the whole hippocampus and the anterior and posterior subdivisions into left- and right-hemisphere ROIs and repeated our analyses in these ROIs.

In the multivariate analyses, multisensory facilitation was driven by the left posterior hippocampus. This region displayed a robust congruent audiovisual effect, with no detectable unisensory similarity in either modality, and significant facilitation under a max criterion. In contrast, crossmodal transfer was right lateralized. This was true both in the anterior hippocampus and at the level of the whole hippocampus.

Importantly, statistical comparisons across hemispheres were not significant for either effect. Thus, the lateralization we describe reflects effects reaching significance within one hemisphere but not the other, rather than a reliable hemispheric difference. With this caveat in mind, the regional patterns are suggestive of a hemispheric dissociation

between facilitation and transfer. They are broadly consistent with accounts of human hippocampal laterality (Burgess et al., 2002; Howard et al., 2014; Maguire et al., 1998; Maguire & Frith, 2003; O'Keefe et al., 1998; Spiers et al., 2001). On these accounts, the left hippocampus is biased toward episodic, detail-rich coding of stimulus content, which stands to benefit from congruent multisensory input. By contrast, the right hippocampus is biased toward coarser, configural representations of spatial structure and statistical dependencies, which are better suited to abstracting across modality-specific detail.

We have included the hemispheric results in the revised main text, after moving the subfields to the supplement. First, we show that visual responses dominate the univariate results in both hemispheres and are not lateralized (Figure 2B):

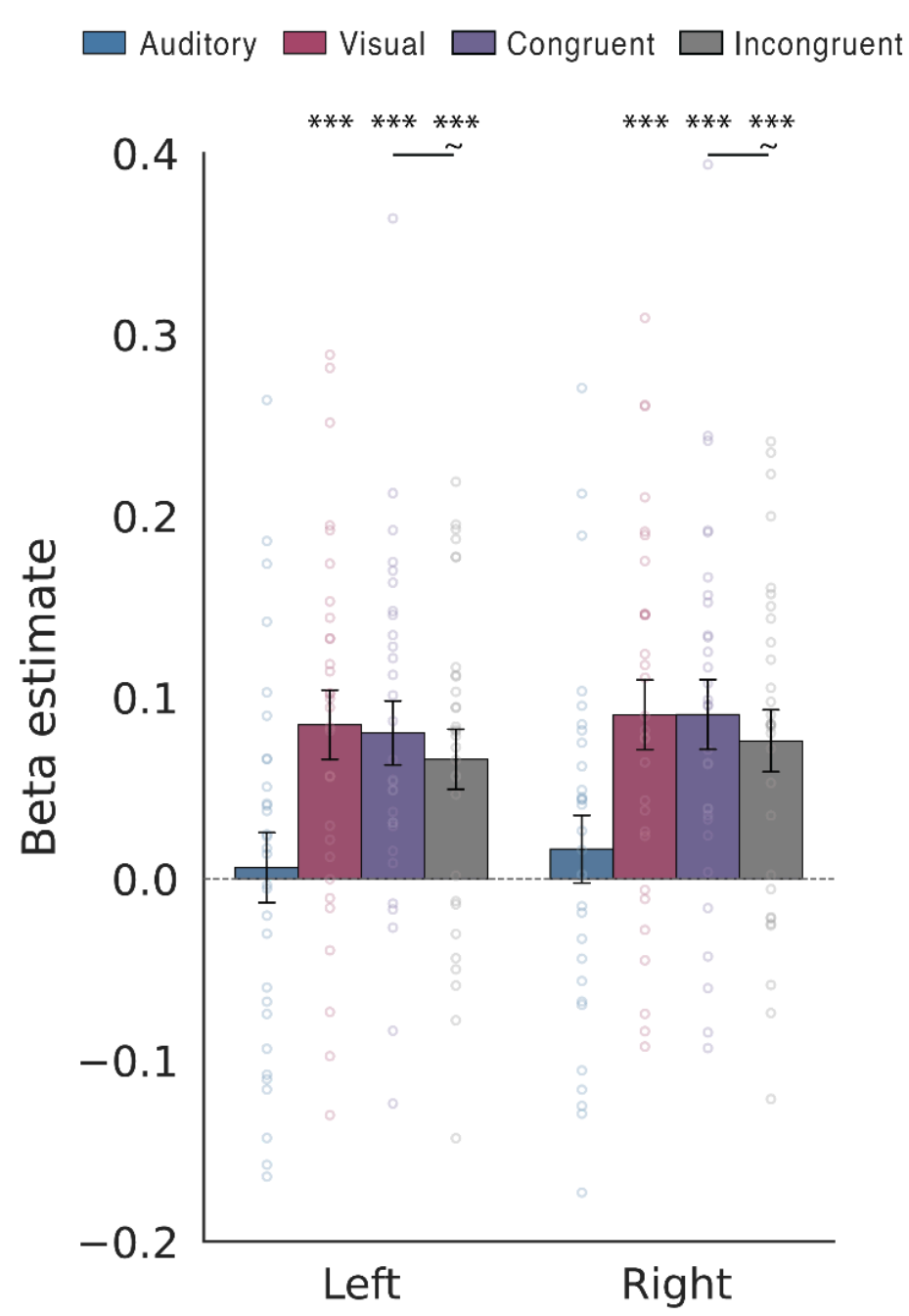

Figure 2: **Univariate activation across hippocampal hemispheres.** Mean beta estimates for each condition in the whole left and right hippocampus. Individual participant data shown as circles. Error bars indicate SEM. ~ $P < 0.1$, * $P < 0.05$, ** $P < 0.01$, *** $P < 0.001$.

Second, we show that multisensory facilitation in the posterior hippocampus (Figure 3C) is driven by the left hemisphere (Figure 3D):

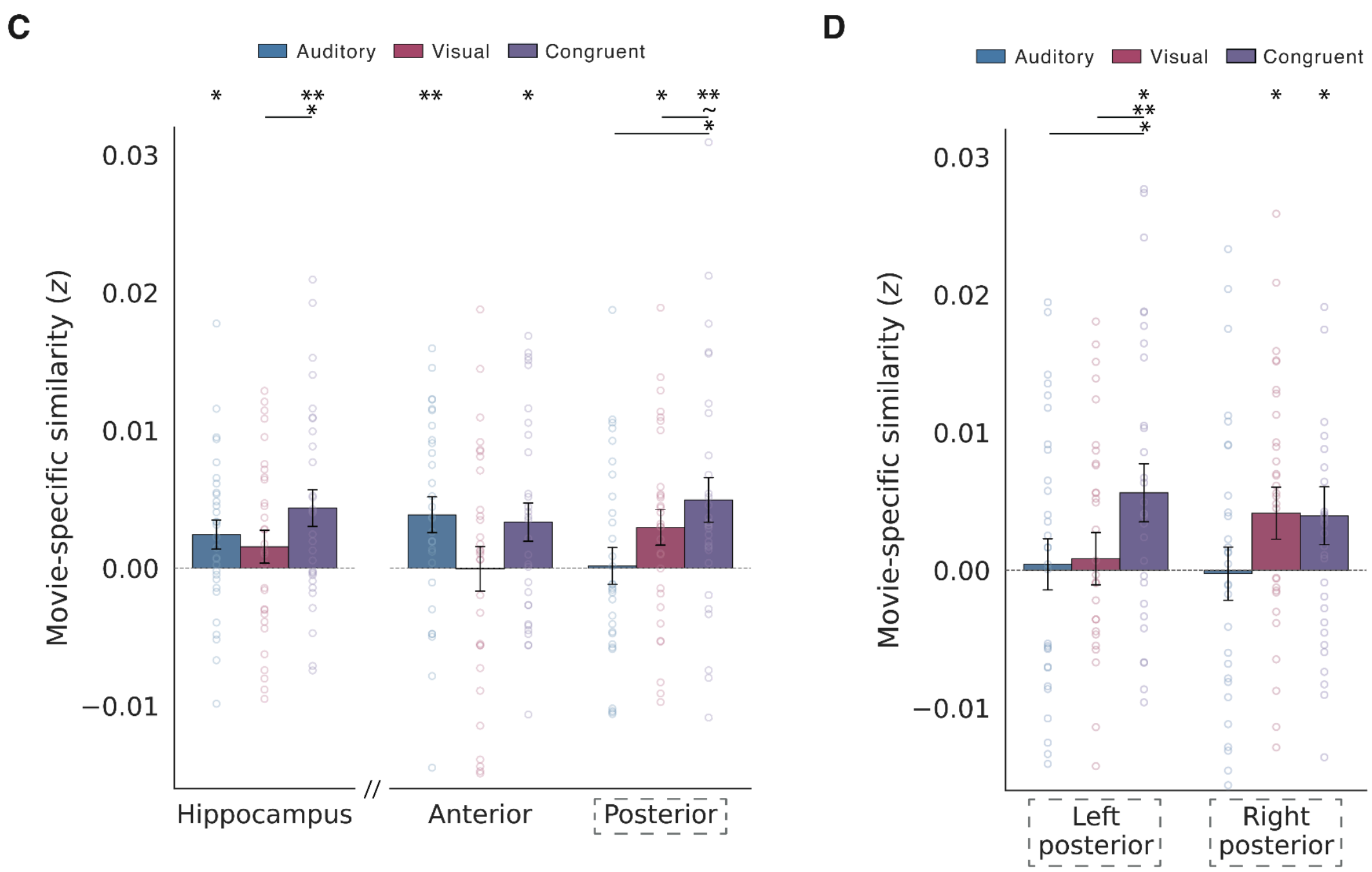

Figure 3: **Movie-specific multivariate representations in the hippocampus. (C)** MSPS across conditions for the whole hippocampus (left) and anterior and posterior subdivisions (right). The posterior hippocampus showed enhanced pattern similarity for congruent audiovisual stimuli relative to unisensory conditions (grey hatched box). **(D)** MSPS across conditions for the left and right posterior hippocampus. Six data points outside the y-axis range are not shown; the range was chosen to match that of panel C to ease comparisons. Individual participant data shown as circles. Error bars indicate SEM. ~ $P < 0.1$, * $P < 0.05$, ** $P < 0.01$, *** $P < 0.001$.

Finally, we show that crossmodal transfer in the anterior hippocampus (Figure 5B) is right-lateralized (Figure 5C):

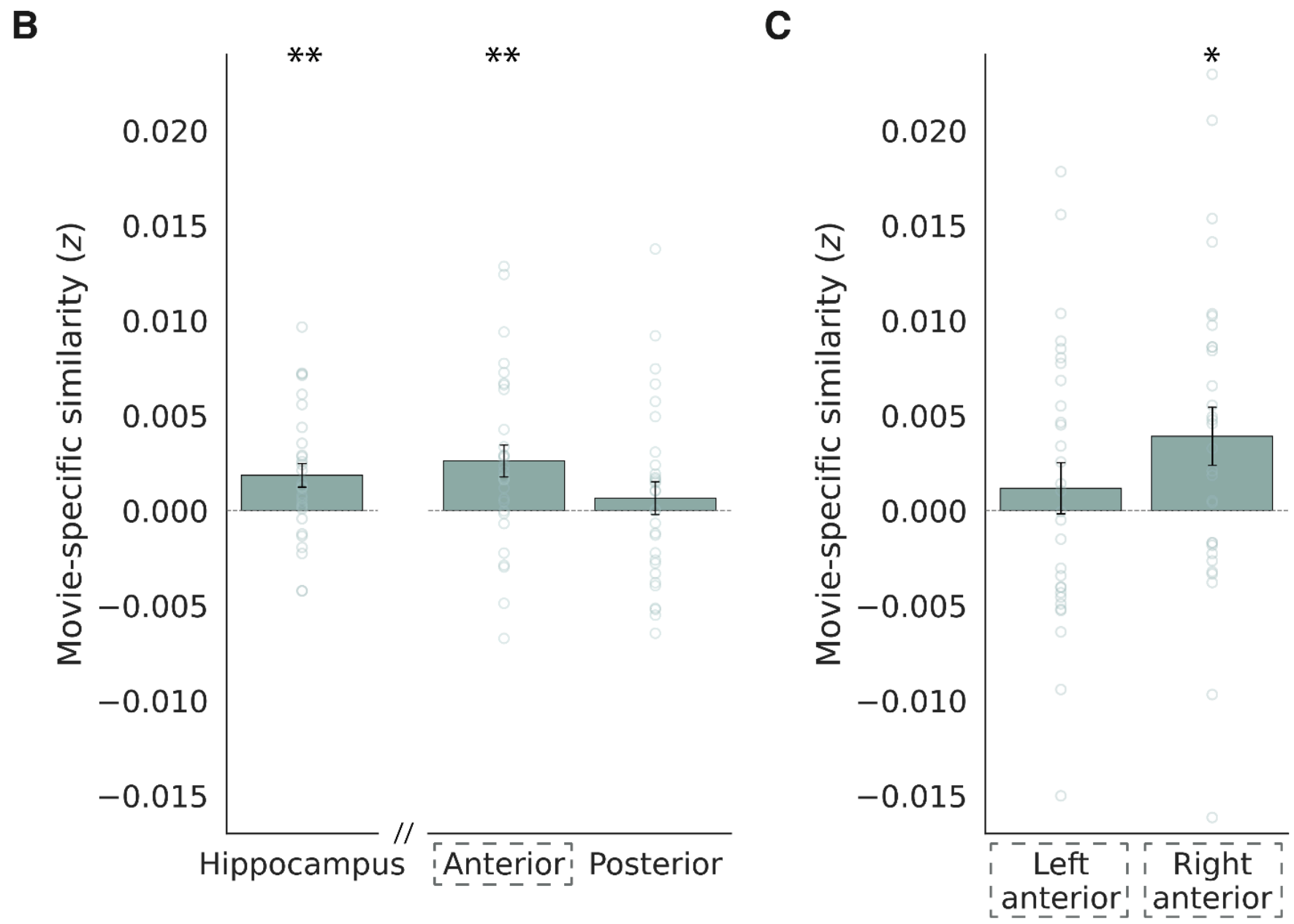

Figure 5: **Crossmodal transfer in the hippocampus. (B)** Crossmodal MSPS in the whole hippocampus andanterior/posterior subdivisions. **(C)** Crossmodal MSPS for the left and right anterior hippocampus. Individual participant data shown as circles; error bars indicate SEM. * *P* < 0.05, ** *P* < 0.01, *** *P* < 0.001.

All remaining lateralization results are reported in Figures S4 and S8. We motivate these findings in the revised Discussion (p. 11-12):

"Our results additionally revealed hemispheric asymmetries along the long axis: multisensory facilitation was strongest in the left posterior hippocampus, whereas crossmodal transfer was driven by the right anterior hippocampus. The human hippocampus displays strong lateralization across a range of memory and navigation tasks (Burgess et al., 2002; Howard et al., 2014; Maguire et al., 1998; Maguire & Frith, 2003; O'Keefe et al., 1998; Spiers et al., 2001). Our results are broadly consistent with a left-hemisphere bias for episodic coding of stimulus content and a right-hemisphere bias for coarser, configural representations that are better suited to abstracting across modality-specific detail. The latter aligns with work showing a right hippocampal bias for statistical learning across different types of visual stimuli (Turk-Browne et al., 2009; Turk-Browne et al., 2010). These interpretations are speculative, as the principles governing hippocampal laterality remain poorly understood. Even so, the asymmetries observed here motivate the use of multisensory stimuli to constrain hypotheses regarding lateralization in the human hippocampus."

Finally, there are two issues in the plotting choices:

In describing the stats, the authors mention using randomization yet they never show these null distributions. I appreciate showing the participant-wise distribution of responses, but visualizing a sense of the noisiness/stability of the effects would also be helpful.
In the MSPS figures, the y axis indicates these are z-scored values. Typically, the figures should be of the "raw" correlations and the stats done on z-scored values. Is there a reason the authors instead chose to plot the z-scored values?

We agree that visualizing the null distributions is valuable. We have added Supplementary Figure S11, which shows the null distributions alongside the observed group-mean statistic for the whole, anterior, and posterior hippocampus, across the main conditions and crossmodal transfer:

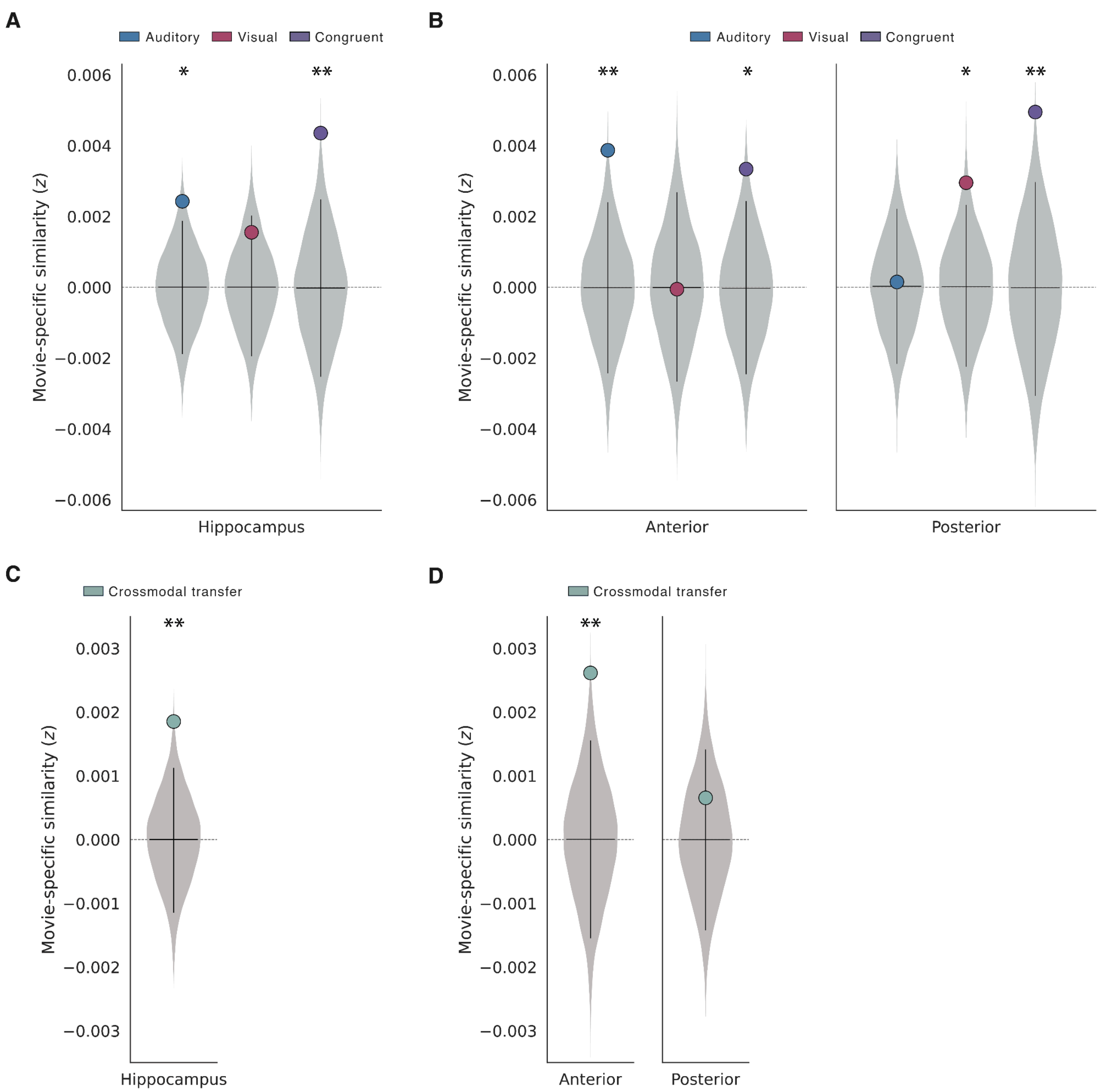

Figure S11: **Permutation null distributions for multivariate effects.** Mean MSPS across participants (colored circles) plotted against the empirical null distributions generated by the same sign-flipping randomization procedure used for statistical testing (10,000 iterations; grey violins). (A) MSPS for the auditory-only, visual-only, and congruent audiovisual conditions in the whole hippocampus. (B) The same, shown separately for the anterior and posterior hippocampus. (C) Crossmodal transfer MSPS in the whole hippocampus. (D) Crossmodal transfer MSPS in the anterior and posterior hippocampus. Grey violins show the kernel density of the null distribution for each ROI and condition. The vertical black bar spans the 5th to 95th percentile of the null and the horizontal black tick marks the null mean. Asterisks denote FDR-corrected significance. * $P < 0.05$, ** $P < 0.01$, *** $P < 0.001$.
