# Supplementary material for: The human hippocampus supports auditory coding and multisensory integration
Source: PLoS Biol. 2026 Nov 3;24(11):e3004012. doi: 10.1371/journal.pbio.3004012 (PMC13730386; doi:10.1371/journal.pbio.3004012)
Supplement: Supplementary file 8 [file pbio.3004012.s021.pdf]

Response to Reviewers

Editor Comments

Based on the reviews and on our Academic Editor's assessment of your revision, we are likely to accept this manuscript for publication, provided you satisfactorily address the remaining points raised by Reviewer 2. We encourage to focus on addressing their points #1 and #2. However, if you would also like to address their point #3 and need more time to do so, please let us know and we'll extend the revision deadline.

Thank you for the opportunity to address these additional suggestions. We have now carefully responded to the three points raised by the reviewer, as detailed below.

We have also updated our manuscript to address the other submission requirements in your action letter. We now include the necessary ethics statement. The raw and preprocessed fMRI data are being deposited to OpenNeuro, and we are uploading CSV files containing the individual data points underlying all main text and supplementary figures to the OSF page. Our task and analysis code are archived on both GitHub and Zenodo. Links to these resources are included in the revised manuscript.

We would like to suggest a different title to improve its accessibility for our broad audience: "Multisensory scene coding reveals functional specialization along the human hippocampal axis"

Thank you for helping to improve the accessibility of our paper. We liked many aspects of your suggestion, but landed on a slight variation, “Functional specialization of multisensory coding in the human hippocampus.”

Reviewer 2 Comments

I thank the authors for considering my comments so carefully and for the thorough revision of the manuscript. I also commend them for running all the suggested additional analyses, the results of which strengthen the manuscript. I have a few comments left that need to be addressed:

We thank the Reviewer for the constructive and thoughtful review of our work.

1. While the results related to the regression coefficients and the proportion of explained variance convincingly suggest that the overlapping representations do not explain the pattern of cross-modal representation in the hippocampus, the results related to the residuals are surprising. It is unclear why the residuals obtained after regressing out the unisensory conditions are above chance level in the sensory regions (while this was expected for the hippocampus)? Shouldn't they be at chance level in the sensory regions? How was chance level computed? I was under the impression that the authors computed the difference between diagonal and off-

diagonal here. Was this difference significant in all ROIs (i.e., was the delta different from zero)?

The authors mention the results of these analyses in the main text for the hippocampus (as they suggest cross-modal representation above and beyond unimodal representations) but omit to report these results for the sensory regions in the main text (as they also suggest representation above the unimodal representation which was not expected). Please include these analyses in the main text and offer an interpretation for the sensory regions as it is unclear why "… residual conjunctive structure was evident in both sensory areas and the hippocampus" (as stated in the revised discussion).

The Reviewer is correct that the residual congruent audiovisual MSPS is computed as the difference between diagonal (within-movie) and off-diagonal (between-movie) values of the cross-validated residual matrix. The chance level for this measure is zero, corresponding to the null hypothesis of no movie-specific similarity. As noted, the residual MSPS was significantly above zero in sensory ROIs (OP, LOC, HG, pSTG). The beta coefficients tracked preferred modality in each region (visual coefficients in OP and LOC; auditory coefficients in HG and pSTG), and the unisensory matrices accounted for substantial variance in the audiovisual matrix (~11–36%).

However, this analysis does not preclude the possibility of movie-specific structure in the residual audiovisual matrices of these ROIs. The unisensory matrices are not a noise-free representation of auditory or visual content; they reflect estimates of neural responses to sensory features from held-out runs. Moreover, the regression approach assumes linearity and there could be non-linear interactions between unisensory and multisensory representations. These limitations of the analysis would explain the above-baseline residual effects as unaccounted unisensory variance. Alternatively, these effects could reflect true conjunctive, audiovisual coding in putative unisensory regions. This would be consistent with the literature on multisensory integration in sensory cortex (Ghazanfar & Schroeder, 2006; Ferraro et al., 2020).

As suggested, we now explicitly note this in the revised Results section (p. 6):

“Finally, the residual congruent audiovisual MSPS remained above baseline in the whole hippocampus ($P = 0.002$), anterior hippocampus ($P = 0.019$), and posterior hippocampus ($P = 0.005$; Figure 4C). Notably, residual congruent audiovisual MSPS was also significantly above zero in the sensory cortical ROIs (all $P < 0.001$; Figure S5).”

We also raise this point in the Discussion section (p. 11):

“Nevertheless, residual conjunctive structure was evident not only in the hippocampus but also in sensory areas. This may indicate that unisensory matrices estimated from held-out runs could not fully account for the unisensory variance in the audiovisual matrix, leaving unexplained unisensory movie-specific structure in the residuals.

Alternatively, these regions could support conjunctive coding of audiovisual inputs, with the residuals reflecting true multisensory movie-specific structure, in line with the role of early sensory regions in multisensory integration (Ghazanfar & Schroeder, 2006; Ferraro et al., 2020)."

2. Pearson correlation can be an unreliable measure of pattern similarity when there are differences in mean univariate activation between conditions (which is expected for the sensory ROIs). How did the authors deal with this potential issue?

As the Reviewer points out, Pearson correlation can result in lower overall estimates of pattern similarity under low SNR (Walther et al., 2016), which could disadvantage non-preferred stimuli (e.g., auditory patterns in visual cortex). Importantly, our main MSPS metric is computed as the difference between within-movie and between-movie similarity separately for each condition. Therefore, any SNR-related attenuation in a non-preferred modality is removed by subtracting the off-diagonal values, isolating movie-specific structure within each condition. Moreover, we do not statistically compare the visual-only and auditory-only conditions, but rather compare congruent audiovisual MSPS to each unisensory condition. Our conclusions therefore do not depend on differences in SNR across modalities.

Nevertheless, we now acknowledge this point in the Discussion section (p. 10):

"Finally, Pearson correlation can be an unreliable measure under conditions of low SNR (Walther et al., 2016), which could attenuate pattern similarity for non-preferred stimuli (e.g., auditory patterns in visual areas). Importantly, our movie-specific similarity metric protects against this concern by baselining against off-diagonal structure within condition."

3. Incongruent matrix: I disagree that the congruent and incongruent matrices can't be compared - as the movie-specific information that carries across runs is unisensory in nature (there is audio-visual incongruency in each run but across runs, there will be audio / visual congruency between movies). In other words, you would expect sensory regions to detect the sensory congruency across runs (and indeed MSPS would not be close to zero in these ROIs) but such unimodal sensory coding is not expected in the "multimodal regions" - or to a lesser extent (as unisensory contribution does not contribute any significant variance to the multimodal condition) which represents a powerful control. I suggest extracting incongruent metrics in the control ROIs and compare the incongruent data to both unisensory and multisensory conditions for all ROIs, including the hippocampus.

We agree with the Reviewer that incongruent MSPS values can carry movie-specific information because they capture modality-specific structure across runs. As such, we reasoned that the unisensory conditions, rather than the congruent condition, provide the most stringent multivariate comparison for the incongruent condition. Namely, the incongruent condition would be expected to show suppression relative to the

unisensory conditions in regions sensitive to multisensory congruence (i.e., non-matching input from a second modality should be worse than no input).

We previously reported this comparison in Figures S6 and S7 for the hippocampal and MTL cortical ROIs, and at the whole-brain level. As suggested by the Reviewer, we have now run these analyses in the sensory cortical ROIs (OP, LOC, HG, pSTG). As expected given prior work, we find robust suppression in pSTG and to a lesser extent in HG. We have updated Figure S6 to include the sensory cortical ROIs:

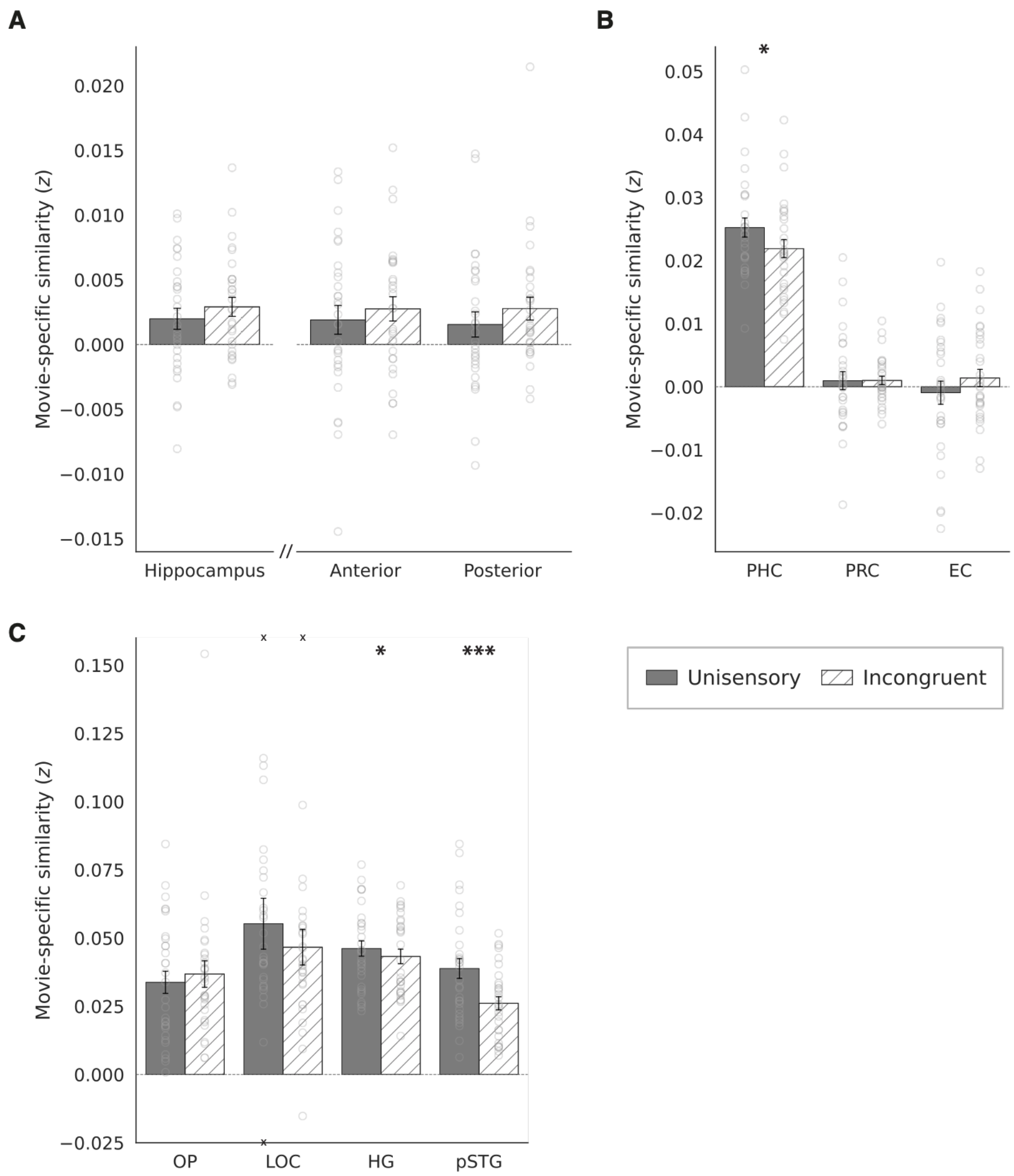

Figure S6: **ROI analysis of unisensory versus incongruent MSPS. (A)** No significant differences were observed between incongruent and unisensory (average of auditory and visual) MSPS in the whole hippocampus and anterior/posterior hippocampus. **(B)** PHC showed greater MSPS for unisensory relative to incongruent conditions, suggesting a destructive interaction between non-matching auditory and visual content. There were no significant differences in PRC or EC. **(C)** Sensory ROIs showed significant suppression in HG and pSTG. Individual participant data shown as circles; error bars indicate SEM. x markers indicate individual data points outside the plotted y-axis range.* $P < 0.05$, *** $P < 0.001$. The data underlying this figure are provided in Main_RSA_ROI_data.csv at osf.io/jcg8a.
