# Supplementary material for: The human hippocampus supports auditory coding and multisensory integration
Source: PLoS Biol. 2026 Nov 3;24(11):e3004012. doi: 10.1371/journal.pbio.3004012 (PMC13730386; doi:10.1371/journal.pbio.3004012)
Supplement: Peer review history

# Editor report: Decision Letter 0

**DOI:** 10.1371/journal.pbio.3004012.r001

**Authors:** Christian Schnell, PhD

6 Mar 2026

Dear Dr Raccah,

Thank you for submitting your manuscript entitled "Multisensory coding of audiovisual movies in the human hippocampus" for consideration as a Research Article by PLOS Biology.

Your manuscript has now been evaluated by the PLOS Biology editorial staff as well as by an academic editor with relevant expertise and I am writing to let you know that we would like to send your submission out for external peer review.

However, before we can send your manuscript to reviewers, we need you to complete your submission by providing the metadata that is required for full assessment. To this end, please login to Editorial Manager where you will find the paper in the 'Submissions Needing Revisions' folder on your homepage. Please click 'Revise Submission' from the Action Links and complete all additional questions in the submission questionnaire.

Once your full submission is complete, your paper will undergo a series of checks in preparation for peer review. After your manuscript has passed the checks it will be sent out for review. To provide the metadata for your submission, please Login to Editorial Manager (https://www.editorialmanager.com/pbiology) within two working days, i.e. by Mar 10 2026 11:59PM.

If your manuscript has been previously peer-reviewed at another journal, PLOS Biology is willing to work with those reviews in order to avoid re-starting the process. Submission of the previous reviews is entirely optional and our ability to use them effectively will depend on the willingness of the previous journal to confirm the content of the reports and share the reviewer identities. Please note that we reserve the right to invite additional reviewers if we consider that additional/independent reviewers are needed, although we aim to avoid this as far as possible. In our experience, working with previous reviews does save time.

If you would like us to consider previous reviewer reports, please edit your cover letter to let us know and include the name of the journal where the work was previously considered and the manuscript ID it was given. In addition, please upload a response to the reviews as a 'Prior Peer Review' file type, which should include the reports in full and a point-by-point reply detailing how you have or plan to address the reviewers' concerns.

During the process of completing your manuscript submission, you will be invited to opt-in to posting your pre-review manuscript as a bioRxiv preprint. Visit http://journals.plos.org/plosbiology/s/preprints for full details. If you consent to posting your current manuscript as a preprint, please upload a single Preprint PDF.

Feel free to email us at plosbiology@plos.org if you have any queries relating to your submission.

Kind regards,

Christian

Christian Schnell, PhD

Senior Editor

PLOS Biology

cschnell@plos.org

# Peer review: Decision Letter 1

**DOI:** 10.1371/journal.pbio.3004012.r002

**Authors:** Christian Schnell, PhD

20 Apr 2026

Dear Dr Raccah,

Thank you for your patience while your manuscript "Multisensory coding of audiovisual movies in the human hippocampus" was peer-reviewed at PLOS Biology. It has now been evaluated by the PLOS Biology editors, an Academic Editor with relevant expertise, and by several independent reviewers.

In light of the reviews, which you will find at the end of this email, we would like to invite you to revise the work to thoroughly address the reviewers' reports.

As you will see below, the reviewers are overall quite positive, but nevertheless raise a few concerns that require additional analyses and textual revisions to expand the discussion of quite a few points.

Given the extent of revision needed, we cannot make a decision about publication until we have seen the revised manuscript and your response to the reviewers' comments. Your revised manuscript is likely to be sent for further evaluation by all or a subset of the reviewers.

In addition to these revisions, you will need to complete some formatting changes, which you will receive in a follow up email. A member of our team will be in touch with a set of requests shortly.

We expect to receive your revised manuscript within 3 months. Please email us (plosbiology@plos.org) if you have any questions or concerns, or would like to request an extension.

At this stage, your manuscript remains formally under active consideration at our journal; please notify us by email if you do not intend to submit a revision so that we may withdraw it.

**IMPORTANT - SUBMITTING YOUR REVISION**

Your revisions should address the specific points made by each reviewer. Please submit the following files along with your revised manuscript:

1. A 'Response to Reviewers' file - this should detail your responses to the editorial requests, present a point-by-point response to all of the reviewers' comments, and indicate the changes made to the manuscript.

*NOTE: In your point-by-point response to the reviewers, please provide the full context of each review. Do not selectively quote paragraphs or sentences to reply to. The entire set of reviewer comments should be present in full and each specific point should be responded to individually, point by point.

You should also cite any additional relevant literature that has been published since the original submission and mention any additional citations in your response.

2. In addition to a clean copy of the manuscript, please also upload a 'track-changes' version of your manuscript that specifies the edits made. This should be uploaded as a "Revised Article with Changes Highlighted" file type.

*Re-submission Checklist*

When you are ready to resubmit your revised manuscript, please refer to this re-submission checklist: https://plos.io/Biology_Checklist

To submit a revised version of your manuscript, please go to https://www.editorialmanager.com/pbiology/ and log in as an Author. Click the link labelled 'Submissions Needing Revision' where you will find your submission record.

Please make sure to read the following important policies and guidelines while preparing your revision:

*Published Peer Review*

Please note while forming your response, if your article is accepted, you may have the opportunity to make the peer review history publicly available. The record will include editor decision letters (with reviews) and your responses to reviewer comments. If eligible, we will contact you to opt in or out. Please see here for more details:

https://blogs.plos.org/plos/2019/05/plos-journals-now-open-for-published-peer-review/

*PLOS Data Policy*

Please note that as a condition of publication PLOS' data policy (http://journals.plos.org/plosbiology/s/data-availability) requires that you make available all data used to draw the conclusions arrived at in your manuscript. If you have not already done so, you must include any data used in your manuscript either in appropriate repositories, within the body of the manuscript, or as supporting information (N.B. this includes any numerical values that were used to generate graphs, histograms etc.). For an example see here: http://www.plosbiology.org/article/info%3Adoi%2F10.1371%2Fjournal.pbio.1001908#s5

*Blot and Gel Data Policy*

We require the original, uncropped and minimally adjusted images supporting all blot and gel results reported in an article's figures or Supporting Information files. We will require these files before a manuscript can be accepted so please prepare them now, if you have not already uploaded them. Please carefully read our guidelines for how to prepare and upload this data: https://journals.plos.org/plosbiology/s/figures#loc-blot-and-gel-reporting-requirements

*Protocols deposition*

To enhance the reproducibility of your results, we recommend that if applicable you deposit your laboratory protocols in protocols.io, where a protocol can be assigned its own identifier (DOI) such that it can be cited independently in the future. Additionally, PLOS ONE offers an option for publishing peer-reviewed Lab Protocol articles, which describe protocols hosted on protocols.io. Read more information on sharing protocols at https://plos.org/protocols?utm_medium=editorial-email&utm_source=authorletters&utm_campaign=protocols

Thank you again for your submission to our journal. We hope that our editorial process has been constructive thus far, and we welcome your feedback at any time. Please don't hesitate to contact us if you have any questions or comments.

Sincerely,

Christian

Christian Schnell, PhD

Senior Editor

PLOS Biology

cschnell@plos.org

------------------------------------

REVIEWS:

Reviewer #1: In this manuscript, Raccah and colleagues examine whether the human hippocampus represents sensory input beyond vision and supports cross-modal representations. After a pre-scanner study phase, a relatively large sample of participants (N=30) viewed short movie clips across four conditions: visual only, auditory only, congruent auditory and visual stimuli, and incongruent auditory and visual stimuli. The authors then analyzed BOLD activity within sub-regions of hippocampus (anterior vs posterior and subfields) using both univariate and multivariate pattern similarity approaches They test whether hippocampal ROIs: 1) represent auditory input, 2) are sensitive to multisensory facilitation, and 3) support cross-modal representations.

They find that univariate responses in the hippocampus are robust for visual stimuli but show no significant modulation for auditory or audio-visual conditions. In contrast, multivariate pattern analyses reveal both auditory and visual representations, with significant cross-modal pattern similarity. This cross-modal similarity is driven by the anterior hippocampus (i.e., not posterior), which shows evidence for crossmodal generalization between auditory and visual stimuli. Overall, this is a great manuscript: well written, technically strong, and focused on a question of broad relevance. The results are compelling, particularly the evidence for cross-modal pattern similarity in the anterior hippocampus. My main suggestions concern interpretation. In particular, the manuscript would benefit from a fuller treatment of retrieval-based alternative accounts of the results, clearer explanation of the control analyses, and more discussion of whether the auditory-only and visual-only conditions may differ in cognitive processes (e.g., imagery, attention) in addition to the input modality itself. Specific comments follow.

Major:

1. Apologies if I missed this, but what, if anything, was presented on the screen during the auditory-only condition?

2. The authors run a control analysis on p5 to determine whether the multivariate results could in principle come from memory retrieval. They write: "The multivariate effects reported above could in principle benefit from memory retrieval, with repeated stimulus exposure allowing for reinstatement of movie-specific details or mental imagery of the absent modality. If so, we would expect that the magnitudes of these effects should increase as stimuli are repeated across cross-validation folds. However, neither multisensory facilitation in the posterior hippocampus (congruent audiovisual >visual MSPS; Spearman's ρ= 0.048, P = 0.45) nor crossmodal transfer in the anterior hippocampus (ρ= 0.05, P = 0.41) strengthened with more stimulus repetition across runs. This lack of correlation suggests that both phenomena were relatively stable and fails to support a retrieval-based account." This is an important issue to address, but the logic of the analysis is not yet clear to me. What exactly was done here? As written, it is difficult to understand how "stimulus repetition across cross-validation folds" was quantified, and why this analysis provides a strong test of a retrieval-based account. A more straightforward approach would be to split the runs in half and ask whether the relevant multivariate effects are stronger in the second half of the experiment than in the first, when repeated exposure should have had more opportunity to strengthen retrieval or imagery-based contributions.

3. Of course, even if such an analysis shows no increase over runs, it would still be possible that retrieval processes were already at ceiling in run 1 because of the pre-scanner study phase. The authors should therefore more fully consider and discuss this alternative account of their findings.

4. Univariate results: Given that a primary goal is to determine whether hippocampal voxels show significant univariate modulation to auditory-only versus video-only movie clips, it is important to clearly establish that any differences between these conditions are driven by input modality rather than by differences in the cognitive processes they engage. Two such processes come to mind. First, imagery or recall. Because the audiovisual clips were paired in memory through both pre-scanner practice and in-scanner presentation, hearing an audio clip alone - for example, the sound of someone cutting a cucumber - may automatically evoke visual imagery of the corresponding scene. The reverse may also occur, with visual input eliciting auditory imagery, but my intuition is that this process may be asymmetric, with auditory input more likely to trigger visual imagery than vice versa. If so, the two conditions may differ not only in sensory modality, but also in the extent to which they recruit internally generated imagery. One possible way to address this may be to ask whether a reactivation index (e.g., % activity during audio-only in V1 vs. visual-only in V1) calculated for each condition is matched. At minimum, the authors should discuss a retrieval-based account of their findings.

5. Second, salience. Could it be that the audio-only and video-only stimuli engage external attention to different degrees, or might one condition be lower in perceptual salience than the other? Relatedly, because participants performed a cover task in which they pressed a button when the stimulus ended, the task may place different attentional-switching demands on the two conditions. For example, if the auditory stimuli are relatively lower in perceptual salience, externally orienting to the task may constitute a larger shift for that condition. Such a possibility could plausibly matter for hippocampal responses, given recent evidence that the hippocampal long axis is functionally differentiated, with posterior hippocampal regions showing stronger links to salience- and target-detection-related processing, whereas anterior regions are more closely associated with networks involved in memory representation (Angeli et al., 2025). Consistent with this concern, the whole-brain analysis appears to show large-scale neocortical differences between the auditory-only and video-only conditions, raising the possibility that these conditions engage different large-scale networks in addition to differing in sensory modality alone. One possible way to address this would be to ask whether, across the 6 s movie period, there are greater univariate responses in dorsal attention or salience-related regions for one condition versus the other, which would help clarify whether the contrast reflects differences in external orienting demands rather than sensory modality per se. A second suggestion is to test whether the button-press reaction times for the two conditions in the cover task are matched.

Minor:

1. Interesting results from cortical MTL structures (parahippocampal cortex, perirhinal cortex, and entorhinal cortex) have been put into the SI, but a full paragraph is given to these results in the Discussion. I'd suggest that authors incorporate these results into the main Results.

2. It seems surprising that there was not a significant univariate response in the auditory condition in any of the hippocampal areas and subfields examined, given that the authors cite work that demonstrate hippocampal responsiveness to a variety of auditory-only tasks. Including some discussion on the lack of univariate auditory-only response, and if that was expected or unexpected would be helpful.

3. Given how the results focus on a dissociation along the long axis of the hippocampus, it'd be valuable to have more discussion of alternative long axis models beyond the coarse to fine gradient, such as anterior encoding vs posterior retrieval (could congruent presentations be more strongly promoting recall?) or spatial versus non-spatial representations (e.g., Poppenk, 2013)

Reviewer #2: This manuscript presents the results of a neuroimaging study investigating cross-modal representations of audio-visual movies in the human hippocampus. The authors used standard univariate analyses and representation similarity analyses of fMRI data acquired in different movie conditions to examine auditory, visual, audio-visual (congruent and incongruent) representations of the movies across the subfields and longitudinal axis of the hippocampus. The results of the RSA showed that the hippocampus represents both auditory and visual scenes. The representation of (congruent) audio-visual scenes was greater than unimodal sensory auditory and visual scenes representations in the posterior hippocampus. Interestingly, patterns were similar across auditory and visual modalities in the anterior hippocampus, suggesting that this region represents scenes irrespective of the modality. The results are discussed in the context of a facilitation / generalization gradient along the hippocampal longitudinal axis. This is a very interesting paper that examines an important research question. I listed below some methodological points that should be addressed to strengthen the paper.

Major points:

-Representation of overlapping conditions

As the same movies were used across all conditions, the audio-visual (congruent) diagonal similarity values could be entirely explained by unimodal sensory coding (ie pure auditory or visual coding). The authors compared similarity values between conditions to test whether e.g. congruent audio-visual coding was greater than unimodal sensory coding. I recommend testing for the contribution of these overlapping representations with e.g., regression analyses to give the readers an idea of how much variance in eg the multimodal condition is explained by unimodal representations. Importantly, it is expected that when the overlapping unimodal representations are regressed out from the multimodal representation, the similarity would still be greater than zero (showing eg multimodal representation that is not fully explained by unimodal representations).

-Additional "control" ROIs

I recommend including additional "unimodal sensory" ROIs that would serve as control ROIs to test for the specific of "primary" sensory coding and e.g. test for visual coding in both a visual and an auditory ROIs, and for auditory coding in both auditory and visual ROIs (and potential facilitation / generalization in these ROIs as well). I also suggest adding "integration" ROIs and test unimodal, multimodal sensory and cross-modal coding in these cortical regions as well. Together with the regressions mentioned above, these additional analyses will allow for a better characterization of the specificity of the results reported in the manuscript. Note that the point above might require moving away from RSA approaches as Pearson correlation can be an unreliable measure of pattern similarity when there are differences in mean univariate activation between conditions (that you could expect between unimodal regressors in these specific ROIs, see Walther et al., 2016).

Also note that the whole brain search light analysis partially addresses this comment (but see point above on potential confound due to differences in univariate activation for unimodal conditions). But it is important to show similarity values for the different conditions (compare them between conditions and provide the regressions mentioned above) to test for the specificity of the hippocampal results.

-Incongruent condition

Please also report the similarity values for the incongruent condition as this would provide a good "surrogate" analysis in which similarity values should be close to zero between diagonal and off-diagonal.

-Familiarization

As participants were familiarized with the stimuli before the scanning session, one could argue that the unimodal sensory conditions might be contaminated by the other modality (the visual only condition of e.g. "the train movie" might trigger auditory imagery and vice versa). The regression analyses mentioned above might help decipher the contribution of unimodal sensory conditions to the effects that are reported (e.g., does the auditory condition trigger visual coding in a visual ROI). It is also important to reflect on such potential limitations in the discussion.

Minor points:

-Performance on the offset detection task: I am not sure I follow what the p values represent here - what is being tested?

"Performance in this offset detection task was high across conditions (auditory: mean accuracy across participants, M = 0.94, standard error of the mean, SEM = 0.006, P < 0.001; visual: M = 0.98, SEM = 0.006, P < 0.001; congruent: M = 0.98, SEM = 0.003, P < 0.001; incongruent: M = 0.99, SEM = 0.003, P < 0.001)."

-What makes a trial "correct"? A response before within the 1s window? Please specify.

-Figure 3A: As the RSA is currently only performed on hippo ROI, I suggest altering the ROI depiction.

-Connectivity analyses can be performed with such event-related design. Therefore, I am not sure I follow the statement below:

"The current event-related design precludes a rigorous analysis of the interactions across these regions, which could be addressed using longer-duration audiovisual stimuli to enable connectivity analyses."

Reviewer #3: This manuscript tackles a fundamental, yet under-explored, question about hippocampal representations regarding dynamic stimuli that vary in their sensory content (visual-only information, auditory-only, coherent audiovisual, incongruent audiovisual). They make the well-taken point that much of the literature (especially within human cognitive neuroscience) has largely focused on visual stimuli (e.g., pictures of objects as used in standard item recognition tasks that proliferate among memory researchers). To investigate the potential broader representational role of MTL, the authors used a clean, well-powered design in which participants viewed (or, in the audio-only condition, listened) to 10 clips in each of the critical conditions, repeated across a 9 runs of fMRI scanning. In the audio, visual, and congruent conditions, the 10 clips remained the same in each run. For the incongruent condition, audio/visual tracks were shuffled such that the 10 clips were a run-unique pairing. This then enabled 1) univariate and 2) multivariate ("movie specific pattern similarity") analyses.

Replicating decades of work, they find univariate HC activity for both visual-only clips and visual + audio (multisensory) clips, irrespective of congruency. Notably, across MTL, the only region to show univariate representations of auditory-only clips was PHC, although this was actually a negative activation (although this is perhaps a bit hard to interpret since I'm assuming they're using the ITI as an implicit baseline). Turning to multivariate representations, they find a quite different pattern of results in which HC seems to demonstrate clip-specific representations for audio-only and congruent audiovisual clips but not visual alone. This perhaps gets at an intriguing distinction between representational vs. mnemonic functions of the HC, although the lack of changes in MSPS across repeated viewings suggests a simple mnemonic explanation may be insufficient.

They additionally find what they frame as a compelling distinction between anterior/postierior HC in which aHC shows elevated within movie similarity regardless of presentation modality (audio-only/visual-only) whereas pHC does not show this "cross-modal transfer." There are additional analysts split by HC subfields, although these do not add substantially. Whole brain findings reveal the "usual" cortical players for unimodal stimuli and results broadly consistent with the nascent but growing naturalistic literature suggesting a role for "posterior medial cortex" in conjunctive coding as well as modality-invariant representation of narratives (Fig 5 C & D).

This paper is well-written with little to no grammatical or linguistic elements that would hinder our understanding. Methods (both stimulus/experimental design) and analytical approach are appropriate. This work follows an emerging trend of asking questions about putative memory structures but without a clear memory component which may present interesting challenges for memory researchers interested in the role of the HC. I did not review the first submission of this paper, but I have the following thoughts on this revision:

The major point I find myself wrestling with is the discrepancy between our interpretation of what HC is doing between the univariate and multivariate analyses (considering also prior literature showing in particular HC representations of visual-only items). Do they think the key difference here is about a viewing-only vs. memory task? This is a very interesting theoretical implication of their work and one I felt was under-specified in the discussion.

Given that this paper is framed as a "first foray," it may be helpful to report the extent of cortical regions (eg, Figs 2, 5, & supplementals) in a supplemental table. In the main figs, they could also consider outlining the extent of brain regions so it's clearer to the reader. Otherwise, it feels a bit like "the naturalistic processing bits of DMN," but I think there may be some important specificity.

Again, perhaps in part because the authors frame the current work as a first pass in an under-explored domain, but, in addition to reporting whole HC and results split by long axis, they additionally report results by HC subfield. To me, there are several problems here: (1) they do not give any theoretical motivation (nor can I see a compelling one) WHY you'd expect differences amongst subfields in this regard. I actually wonder if instead the subfield findings should be in the supplemental. And 2) given their subfield results, it is sometimes challenging to reason about how their long axis findings could arise. For instance, in Fig 4, given that much of aHC is comprised of CA1 finding MSPS that is indistinguishable from zero, how can it be that aHC shows a strong MSPS effect in panel B?

Moving beyond subfields, I would have liked to see results either split by hemisphere or, better yet, hemisphere statistically compared. HC exhibits other laterality effects and so it could help provide a helpful baseline expectation for future work in this domain.

Finally, there are two issues in the plotting choices:

In describing the stats, the authors mention using randomization yet they never show these null distributions. I appreciate showing the participant-wise distribution of responses, but visualizing a sense of the noisiness/stability of the effects would also be helpful.

In the MSPS figures, the y axis indicates these are z-scored values. Typically, the figures should be of the "raw" correlations and the stats done on z-scored values. Is there a reason the authors instead chose to plot the z-scored values?

# Author comment: Author response to Decision Letter 2

**DOI:** 10.1371/journal.pbio.3004012.r003

21 Jul 2026

Attachment Submitted filename: Reply_to_Reviewers.pdf

# Peer review: Decision Letter 2

**DOI:** 10.1371/journal.pbio.3004012.r004

**Authors:** Christian Schnell, PhD

24 Aug 2026

Dear Dr Raccah,

Thank you for your patience while we considered your revised manuscript "Multisensory coding of audiovisual movies in the human hippocampus" for publication as a Research Article at PLOS Biology. This revised version of your manuscript has been evaluated by the PLOS Biology editors, the Academic Editor and one of the original reviewers.

Based on the reviews and on our Academic Editor's assessment of your revision, we are likely to accept this manuscript for publication, provided you satisfactorily address the remaining points raised by Reviewer 2. We encourage to focus on addressing their points #1 and #2. However, if you would also like to address their point #3 and need more time to do so, please let us know and we'll extend the revision deadline.

Please also make sure to address the following data and other policy-related requests:

* We would like to suggest a different title to improve its accessibility for our broad audience: "Multisensory scene coding reveals functional specialization along the human hippocampal axis"

* Please include information in the Methods section whether the study has been conducted according to the principles expressed in the Declaration of Helsinki.

* DATA POLICY:

You may be aware of the PLOS Data Policy, which requires that all data be made available without restriction: http://journals.plos.org/plosbiology/s/data-availability. For more information, please also see this editorial: http://dx.doi.org/10.1371/journal.pbio.1001797

Note that we do not require all raw data. Rather, we ask that all individual quantitative observations that underlie the data summarized in the figures and results of your paper be made available in one of the following forms:

1) Supplementary files (e.g., excel). Please ensure that all data files are uploaded as 'Supporting Information' and are invariably referred to (in the manuscript, figure legends, and the Description field when uploading your files) using the following format verbatim: S1 Data, S2 Data, etc. Multiple panels of a single or even several figures can be included as multiple sheets in one excel file that is saved using exactly the following convention: S1_Data.xlsx (using an underscore).

2) Deposition in a publicly available repository. Please also provide the accession code or a reviewer link so that we may view your data before publication.

Regardless of the method selected, please ensure that you provide the individual numerical values that underlie the summary data displayed in the following figure panels as they are essential for readers to assess your analysis and to reproduce it: 2AB, 3CD, 4BC, 5BC, S1BC, S2AB, S3AB, S4AB, S5ABC, S6AB, S8AB, S9AB, S10AB and S11ABCD.

NOTE: the numerical data provided should include all replicates AND the way in which the plotted mean and errors were derived (it should not present only the mean/average values).

Please also ensure that figure legends in your manuscript include information on where the underlying data can be found, and ensure your supplemental data file/s has a legend.

Please ensure that your Data Statement in the submission system accurately describes where your data can be found.

* CODE POLICY

Per journal policy, if you have generated any custom code during the course of this investigation, please make it available without restrictions. Please ensure that the code is sufficiently well documented and reusable, and that your Data Statement in the Editorial Manager submission system accurately describes where your code can be found. More information on our Code Policy, what and how to share can be found here: https://journals.plos.org/plosbiology/s/code-availability

Please note that we cannot accept sole deposition of code in GitHub, as this could be changed after publication. However, you can archive this version of your publicly available GitHub code to Zenodo. Once you do this, it will generate a DOI number, which you will need to provide in the Data Accessibility Statement (you are welcome to also provide the GitHub access information). See the process for doing this here: https://docs.github.com/en/repositories/archiving-a-github-repository/referencing-and-citing-content

As you address these items, please take this last chance to review your reference list to ensure that it is complete and correct. If you have cited papers that have been retracted, please include the rationale for doing so in the manuscript text, or remove these references and replace them with relevant current references. Any changes to the reference list should be mentioned in the cover letter that accompanies your revised manuscript.

In addition to these revisions, you may need to complete some formatting changes, which you will receive in a follow up email. A member of our team will be in touch with a set of requests shortly. If you do not receive a separate email within a few days, please assume that checks have been completed, and no additional changes are required.

We expect to receive your revised manuscript within two weeks.

To submit your revision, please go to https://www.editorialmanager.com/pbiology/ and log in as an Author. Click the link labelled 'Submissions Needing Revision' to find your submission record. Your revised submission must include the following:

- a cover letter that should detail your responses to any editorial requests, if applicable, and whether changes have been made to the reference list

- a Response to Reviewers file that provides a detailed response to the reviewers' comments (if applicable, if not applicable please do not delete your existing 'Response to Reviewers' file.)

- a track-changes file indicating any changes that you have made to the manuscript.

NOTE: If Supporting Information files are included with your article, note that these are not copyedited and will be published as they are submitted. Please ensure that these files are legible and of high quality (at least 300 dpi) in an easily accessible file format. For this reason, please be aware that any references listed in an SI file will not be indexed. For more information, see our Supporting Information guidelines:

https://journals.plos.org/plosbiology/s/supporting-information

*Published Peer Review History*

Please note that you may have the opportunity to make the peer review history publicly available. The record will include editor decision letters (with reviews) and your responses to reviewer comments. If eligible, we will contact you to opt in or out. Please see here for more details:

https://plos.org/published-peer-review-history/

*Press*

Should you, your institution's press office or the journal office choose to press release your paper, please ensure you have opted out of Early Article Posting on the submission form. We ask that you notify us as soon as possible if you or your institution is planning to press release the article.

*Protocols deposition*

To enhance the reproducibility of your results, we recommend that if applicable you deposit your laboratory protocols in protocols.io, where a protocol can be assigned its own identifier (DOI) such that it can be cited independently in the future. Additionally, PLOS ONE offers an option for publishing peer-reviewed Lab Protocol articles, which describe protocols hosted on protocols.io. Read more information on sharing protocols at https://plos.org/protocols?utm_medium=editorial-email&utm_source=authorletters&utm_campaign=protocols

Please do not hesitate to contact me should you have any questions.

Sincerely,

Christian

Christian Schnell, PhD

Senior Editor

cschnell@plos.org

PLOS Biology

------------------------------------------------------------------------

Reviewer remarks:

Reviewer #2: I thank the authors for considering my comments so carefully and for the thorough revision of the manuscript. I also commend them for running all the suggested additional analyses, the results of which strengthen the manuscript. I have a few comments left that need to be addressed:

1.While the results related to the regression coefficients and the proportion of explained variance convincingly suggest that the overlapping representations do not explain the pattern of cross-modal representation in the hippocampus, the results related to the residuals are surprising. It is unclear why the residuals obtained after regressing out the unisensory conditions are above chance level in the sensory regions (while this was expected for the hippocampus)? Shouldn't they be at chance level in the sensory regions? How was chance level computed? I was under the impression that the authors computed the difference between diagonal and off-diagonal here. Was this difference significant in all ROIs (i.e., was the delta different from zero)?

The authors mention the results of these analyses in the main text for the hippocampus (as they suggest cross-modal representation above and beyond unimodal representations) but omit to report these results for the sensory regions in the main text (as they also suggest representation above the unimodal representation which was not expected). Please include these analyses in the main text and offer an interpretation for the sensory regions as it is unclear why "… residual conjunctive structure was evident in both sensory areas and the hippocampus" (as stated in the revised discussion).

2.Pearson correlation can be an unreliable measure of pattern similarity when there are differences in mean univariate activation between conditions (which is expected for the sensory ROIs). How did the authors deal with this potential issue?

3.Incongruent matrix: I disagree that the congruent and incongruent matrices can't be compared - as the movie-specific information that carries across runs is unisensory in nature (there is audio-visual incongruency in each run but across runs, there will be audio / visual congruency between movies). In other words, you would expect sensory regions to detect the sensory congruency across runs (and indeed MSPS would not be close to zero in these ROIs) but such unimodal sensory coding is not expected in the "multimodal regions" - or to a lesser extent (as unisensory contribution does not contribute any significant variance to the multimodal condition) which represents a powerful control. I suggest extracting incongruent metrics in the control ROIs and compare the incongruent data to both unisensory and multisensory conditions for all ROIs, including the hippocampus.

# Author comment: Author response to Decision Letter 3

**DOI:** 10.1371/journal.pbio.3004012.r005

31 Aug 2026

Attachment Submitted filename: Response_Letter.pdf

# Editor report: Decision Letter 3

**DOI:** 10.1371/journal.pbio.3004012.r006

**Authors:** Christian Schnell, PhD

9 Sep 2026

Dear Omri,

Thank you for the submission of your revised Research Article "Functional specialization of multisensory coding in the human hippocampus" for publication in PLOS Biology. On behalf of my colleagues and the Academic Editor, Raphael Kaplan, I am pleased to say that we can in principle accept your manuscript for publication, provided you address any remaining formatting and reporting issues. These will be detailed in an email you should receive within 2-3 business days from our colleagues in the journal operations team; no action is required from you until then. Please note that we will not be able to formally accept your manuscript and schedule it for publication until you have completed any requested changes.

When you attend to these requests, please remember to update the title and Data Availability statement as discussed via email.

Please take a minute to log into Editorial Manager at http://www.editorialmanager.com/pbiology/, click the "Update My Information" link at the top of the page, and update your user information to ensure an efficient production process.

PRESS

We frequently collaborate with press offices. If your institution or institutions have a press office, please notify them about your upcoming paper at this point, to enable them to help maximize its impact. If the press office is planning to promote your findings, we would be grateful if they could coordinate with biologypress@plos.org. If you have previously opted in to the early version process, we ask that you notify us immediately of any press plans so that we may opt out on your behalf.

We also ask that you take this opportunity to read our Embargo Policy regarding the discussion, promotion and media coverage of work that is yet to be published by PLOS. As your manuscript is not yet published, it is bound by the conditions of our Embargo Policy. Please be aware that this policy is in place both to ensure that any press coverage of your article is fully substantiated and to provide a direct link between such coverage and the published work. For full details of our Embargo Policy, please visit http://www.plos.org/about/media-inquiries/embargo-policy/.

Thank you again for choosing PLOS Biology for publication and supporting Open Access publishing. We look forward to publishing your study.

Sincerely,

Christian

Christian Schnell, PhD,

Senior Editor

PLOS Biology

cschnell@plos.org
